# Does the Polypill Improve Patient Adherence Compared to Its Individual Formulations? A Systematic Review

**DOI:** 10.3390/pharmaceutics12020190

**Published:** 2020-02-22

**Authors:** Ana Baumgartner, Katarina Drame, Stijn Geutjens, Marja Airaksinen

**Affiliations:** 1 Division of Pharmacology and Pharmacotherapy, Faculty of Pharmacy, University of Helsinki, 00014 Helsinki, Finland; katarina.drame@gmail.com (K.D.); stijngeutjens@gmail.com (S.G.); marjaairaksinen@gmail.com (M.A.); 2 Faculty of Pharmacy, University of Ljubljana, 1000 Ljubljana, Slovenia; 3 Faculty of Pharmaceutical Sciences, Katholieke Universiteit Leuven, 3000 Leuven, Belgium

**Keywords:** polypill, fixed-dose combination, adherence, systematic review

## Abstract

Many patients, especially those with a high pill burden and multiple chronic illnesses, are less adherent to medication. In medication treatments utilizing polypills, this problem might be diminished since multiple drugs are fused into one formulation and, therefore, the therapy regimen is simplified. This systematic review summarized evidence to assess the effect of polypills on medication adherence. The following databases were searched for articles published between 1 January 2000, and 14 May 2019: PubMed, Web of Science, Cochrane Library, and Scopus. Medication adherence was the only outcome assessed, regardless of the method of measuring it. Sixty-seven original peer-reviewed articles were selected. Adherence to polypill regimens was significantly higher in 56 articles (84%) compared to multiple pill regimens. This finding was also supported by the results of 13 out of 17 selected previously published systematic reviews and meta-analyses dealing with this topic. Adherence can be improved through the formulation of polypills, which is probably why the interest in researching them is growing. There are many polypills on the market, but the adherence studies so far focused mainly on a small range of medical conditions.

## 1. Introduction

Poor medication adherence is a widespread and unresolved challenge among patients [1]. Only half of the prescribed doses are taken, and many patients stop their treatment before the planned end of the therapy [1,2,3]. Several factors contribute to low adherence rates, such as ineffective communication between the patient and the physician, or patients perceive their treatment as unnecessary. Patients may also think the benefits of their pharmacotherapy do not outweigh its adverse effects, or they simply forget [4]. This very often results in complications, extra healthcare costs, side effects, and therapeutic failures. Therefore, improving adherence is a crucial factor in increasing the likelihood of positive therapeutic outcomes.

Patients with chronic illnesses must quite often take multiple pills every day for months or even years, which will eventually result in less adherence to their medication [5]. This occurs especially in cardiovascular diseases (CVD), where patients do not feel the symptoms of their disease in the short term, and it is easier for them to forget to take their medicines [6]. The same goes for diabetes patients; in mild forms, diabetes does not cause serious complications, and patients do not feel ill; thus, they tend to forget their medication [7].

Polypills are a technological innovation that is expected to improve adherence by simplifying the pharmacotherapy regimen [2,5]. The concept of the polypill, very often referred to as a fixed-dose combination (FDC), is quite simple. Instead of taking two or more pills (each containing one active ingredient), multiple drugs are combined into one formulation [6,8,9]. It is generally thought that taking fewer pills will lead to better adherence [2]. This systematic review examines the evidence for that idea and assesses the evidence of the effects of a reduced pill burden on medication adherence.

## 2. Materials and Methods

### 2.1. Search Strategy

This systematic review is focused on articles concerning fixed-dose combinations (FDCs), also known as polypills, in comparison to their separate drug formulations (multiple tablets, free-pill combinations). It does not matter how many drugs are combined in a certain formulation.

The method followed the Preferred Reporting Items for Systematic Reviews and Meta-Analyses (PRISMA) statement [10]. The search was done in May 2019, and it covered the following databases: PubMed, Web of Science, Cochrane Library, and Scopus. After screening all titles and articles, the reference lists of selected articles were used to identify additional relevant studies.

In all four databases, the following selection of keywords was applied: (compliance OR adherence OR non-compliance OR non-adherence OR noncompliance OR nonadherence) AND ((fixed NEAR/1 combination*) OR single-pill* OR single-tablet* OR polypill* OR “combination pill*”). The principle behind this selection was to make an extensive search that would cover only the relevant articles by using as many synonyms and antonyms for two terms related to the aim of our study: polypills and adherence. The search included a language filter, which showed only articles written in English. Furthermore, a time-span filter was used, which included only articles published since 1 January 2000.

### 2.2. Inclusion and Exclusion Criteria

Articles were included in the systematic review if they were either original peer-reviewed studies or systematic reviews and meta-analyses. Narrative reviews and conference abstracts were excluded. There were no restrictions concerning the type of patients, diseases, comorbidities, or drugs. Adherence was the only essential outcome measure for an article to be considered, regardless of how it was measured. Other outcomes were not assessed. All articles needed to have a comparison between low and high pill burden groups, meaning that one group had to take more pills than another group. This was possible either with control groups (longitudinal, controlled) or when observing one group with patients who changed their pill burden over time (longitudinal, uncontrolled). It was also necessary that the articles dealt with solid dosage formulations rather than with any other dosage form.

Additionally, articles dealing with persistence instead of adherence were excluded. The definitions of these two terms are vague since they stand for a similar phenomenon and, thus, they tend to overlap in different literature. However, for this study, only articles measuring adherence were included, and the ones that clearly stated that they dealt only with persistence were excluded.

### 2.3. Study Selection and Data Extraction

Both researchers (A.B. and K.D.) searched for the articles separately to make the most credible and objective article selection. Their findings were then compared, and discussions were held until a final decision about included articles was reached.

Key information about all relevant studies was extracted from the articles. For original peer-reviewed studies, the extracted information included author of the study, year of publication, study country, design, setting, aim and population, disease in question, follow-up period, adherence measures, main outcomes, number and international nonproprietary names (INN) of active ingredients used in the study, and their dosage (if given). For systematic reviews and meta-analyses, the extracted data covered the author of the study, year of publication, medical condition in focus, study aim, number of included original studies, and main results. For further working progress, articles were grouped by (i) article type, that is, original studies separately from systematic reviews and meta-analyses, and (ii) the type of disease they were dealing with.

### 2.4. Quality Assessment

Quality assessment of the included studies was systematically done using Cochrane Collaboration’s risk of bias assessment tool for randomized controlled trials [11] and the Newcastle–Ottawa scale for cohort studies [12]. As adherence was the only outcome of interest, assessment of how any other outcomes were dealt with was ignored.

Cochrane Collaboration’s risk of bias tool measures risk of bias in randomized controlled trials through seven domains: sequence generation and allocation concealment (selection bias), blinding of participants (performance bias), blinding of outcome assessment (detection bias), incomplete outcome data (attrition bias), selective reporting (reporting bias), and other bias. Each domain is assessed as having high, low, or unclear risk of bias. Studies with low risk of bias for all criteria were considered to be of low risk, studies with low or unclear risk of bias were considered medium risk, and studies with at least one domain assessed as high risk of bias were considered as having an overall high risk of bias [11]. However, as all randomized controlled trials were inherently open-label, meaning that blinding of participants was impossible due to the nature of the intervention, this domain was always assessed as having a high risk of bias and, therefore, omitted from the overall evaluation of studies.

The Newcastle-Ottawa scale was used for observational studies; it measures quality through three main domains: selection, comparability, and outcome. Each study was awarded a maximum of nine stars, depending on whether it reached certain standards within these domains (maximum four stars for selection, two stars for comparability, and three stars for outcome) [12]. Studies with 0–4 stars were considered as low quality, studies with 5–7 stars were considered as medium quality, and studies having eight or nine stars were considered as high quality.

## 3. Results

**Figure 1:**
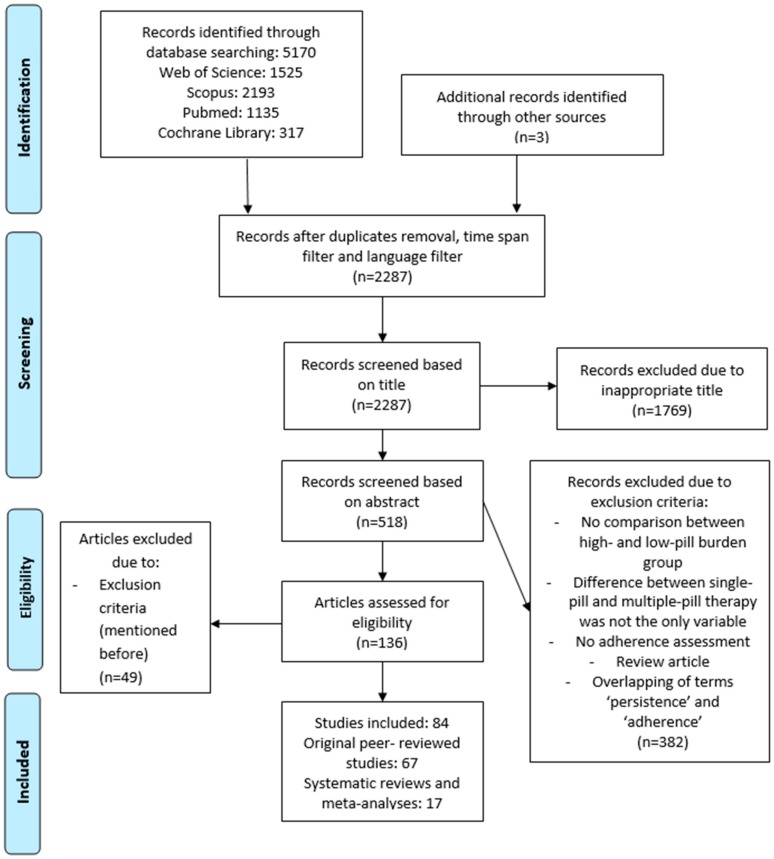
Preferred Reporting Items for Systematic Reviews and Meta-Analyses (PRISMA) flow chart of the article selection.

The database search yielded 5170 records, of which 2287 were screened after removal of duplicates and inclusion of time-span and language filters. After the inclusion and exclusion criteria were applied to screen the records, 84 articles were included in our systematic review; 67 of them were original peer-reviewed studies and 17 were systematic reviews and/or meta-analyses. For the flow chart of the article selection process, see Figure 1.

### 3.1. Included Systematic Reviews and Meta-Analyses (n = 17)

After the selection process of eligible studies, 17 systematic reviews and/or meta-analyses out of 136 articles were included in this systematic review (Figure 1, Table A1, Appendix A). Of these studies, eight were meta-analyses (47%) [13,14,15,16,17,18,19,20], two were systematic reviews (12%) [21,22], and seven were defined as a systematic review with meta-analysis (41%) [23,24,25,26,27,28,29]. The most common systematically reviewed medical condition was hypertension (*n* = 5, 29%) [14,15,16,25,26], followed by studies dealing with CVDs in general (*n* = 3, 18%) [17,18,21], human immunodeficiency virus (HIV) (*n* = 4, 24%) [19,20,24,27], diabetes (*n* = 2, 12%) [22,28], and tuberculosis (*n* = 1, 6%) [23] (Table 1). Two studies examined the effect of polypills in several medical conditions [13,29]. Thirteen of the selected articles (76%) favored therapy with FDC over separate-pill therapy regimens [13,14,15,16,18,19,20,21,22,24,25,27,28].

**Figure 2:**
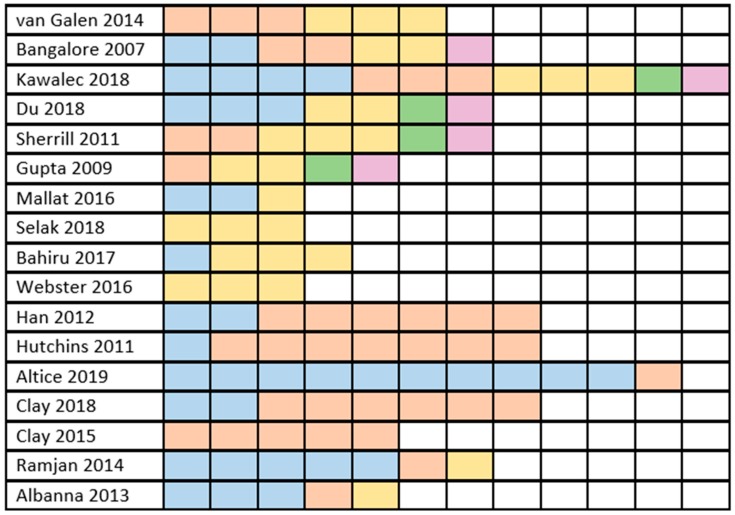
Visual representation of the overlap of the studies included in other systematic reviews and meta-analyses (SR and MA; *n* = 17). One row represents one SR/MA. Each colored square symbolizes one article, and the number of colored squares is equal to the number of studies included in the corresponding SR/MA. Different colors represent into how many SRs/MAs an article was included (e.g., if all the colored squares in a row are blue, all the articles are unique to only this SR/MA). Blue: study included only in one SR/MA. Red: study included in two different SRs/MAs. Yellow: study included in three different SRs/MAs. Green: study included in four different SRs/MAs. Purple: study included in five different SRs/MAs.

However, some overlap of the articles that these studies investigated was found. For example, Selak et al. [17] and Webster et al. [18] included the same studies, which were also included by Bahiru et al. [21] Furthermore, there is much overlap between studies conducted by Gupta et al. [16], Kawalec et al. [25], Sherrill et al. [15], and Du et al. [14]. Both studies conducted by Clay et al. [20,24] share some of the included articles as well. For a visual representation of the overlap of the studies included in the abovementioned systematic reviews and meta-analyses, please see Figure 2.

Some of the studies investigated in one or more of the previously published systematic reviews and meta-analyses were also included in our systematic review since they fit the inclusion criteria [5,30,31,32,33,34,35,36,37,38,39,40,41,42,43,44,45,46,47,48,49,50,51,52,53,54,55,56,57].

### 3.2. Included Original Peer-Reviewed Studies (n = 67)

Altogether, 67 original peer-reviewed studies out of a total of 5170 articles met the inclusion criteria and were included in our study (Figure 1). Of the 67 articles, 31 (46%) were related to hypertension (HT), 14 (21%) were related to human immunodeficiency virus (HIV), 11 (16%) were related to cardiovascular disease (CVD), 10 (15%) were related to diabetes mellitus type II (DMII), and one dealt with lower urinary tract symptoms associated with benign prostatic hyperplasia (LUTS/BHP). Studies were conducted in different countries worldwide; some of them even included more than one country. Most of them (*n* = 36) were conducted in the United States, and only one was carried out in South America, in two different countries. More details can be found in Table 2.

A summary of these 67 studies can be found in Table A2 (Appendix B), displaying author, year of publication, country of the study, study design, study aim, study setting and follow-up period, study population, outcome measures, and results. 

Most of the studies (*n* = 41; 61%) examined the effects of treatment with polypills, where only two drugs were combined (see Table 2 and Table 3). However, in HIV therapy, the use of three-drug formulations was dominant over any other (seven out of 14 studies). There were no data on combining more than five drugs into one formulation. Additional information about the combinations of active ingredients in polypills can be found in Table A3 (Appendix C).

### 3.3. Adherence Measures Used in the Studies (n = 67)

Table 4 summarizes methods for measuring adherence that were used in the selected articles (*n* = 67). Most of the studies (*n* = 62; 93%) relied only on one method; however, five studies [35,50,59,79,82] combined two different methods to assess medication adherence. The applied methods could be divided into two broad categories: subjective (e.g., patient interviews and self-reporting) and indirect (e.g., pill counts, methods using prescription fills, electronic monitoring) [75]. Some of the methods are more general and applicable to more cases, whereas some were used only in a specific study. The most commonly used measure was medical possession ratio (MPR; *n* = 30, 45%), followed by proportion of days covered (PDC; *n* = 21, 31%).

### 3.4. Adherence Outcome

All studies had one or more groups that received more pills than their control groups (Table A2, Appendix B). Those groups could be either a cohort of the same group or a comparison between two different groups. In most cases, the control group was a group of patients on the usual therapy (multiple pills). The test subjects received exactly the same active ingredients as the control group, but in a single formulation [31,32,33,35,41,42,43,54,59,61,67,69,70,73,81,85,89]; alternatively, the test group and the control group were not necessarily receiving the same drugs, but they simply had a different pill burden [5,30,34,36,37,38,39,40,44,45,46,47,48,49,50,51,52,53,55,56,57,58,60,62,64,65,66,68,71,72,74,75,76,77,78,79,80,82,83,84,86,87,88,90,91,92,93,94,95].

The main interest of this review is how the pill burden is associated with patient adherence. In 56 out of 67 studies (84%), there was a significant difference in adherence between the test and control group (Table 5). In seven studies (10%), the difference between both groups was insignificant. In only two studies (3%), both opposite outcomes (improved and decreased adherence in the test group, depending on the treatment situation before the study index date) were reported [61,80]. See Table 5 for a summary of the outcomes.

### 3.5. Quality Assessment of the Included Studies

**Figure 3:**
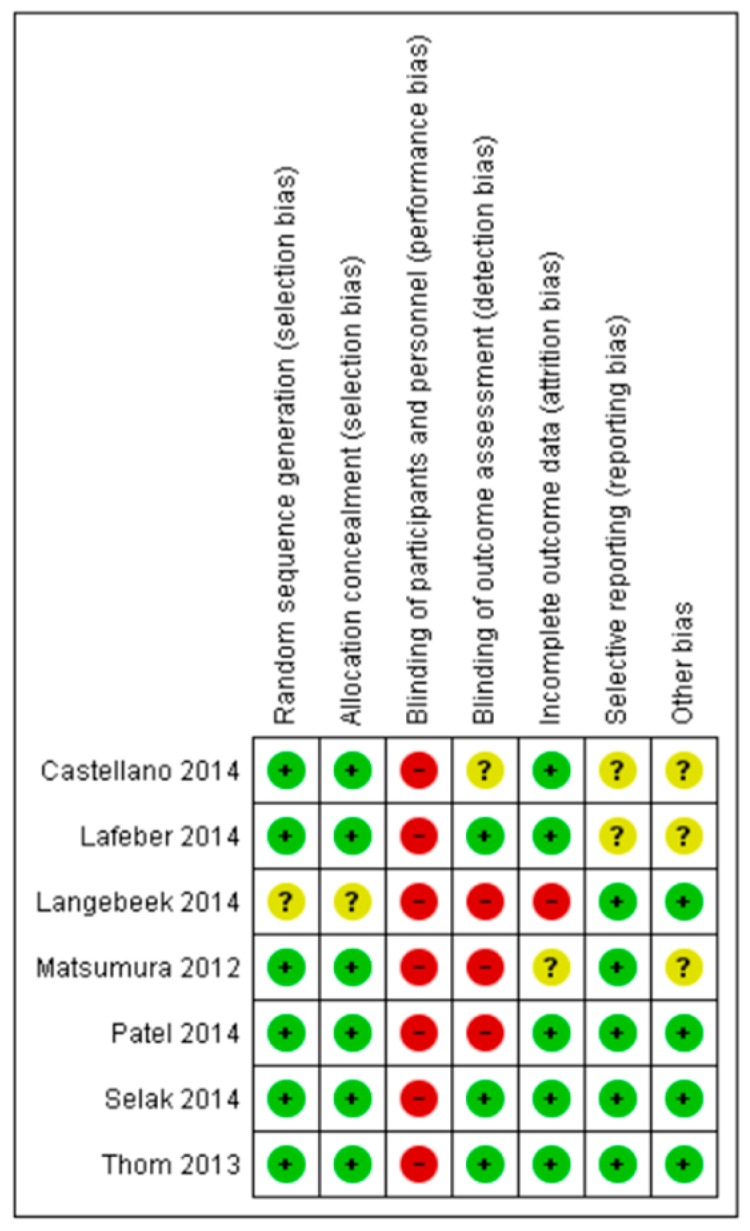
Results of risk of bias assessment for all randomized controlled trials (RCTs). Green: low risk of bias; red: high risk of bias; yellow: unclear risk of bias.

Figure 3 shows the risk of bias summary for all seven randomized controlled trials [35,45,48,52,59,75,88]. As previously noted, blinding of participants was impossible due to the nature of the intervention, that is, different pill burden, resulting in high risk of performance bias in all studies. Thus, it was decided to be omitted from the overall risk of bias assessment. Based on the previously determined criteria, two RCTs reached standards for having an overall low risk of bias [48,52], two studies reached standards for having a medium risk of bias [35,59], and three studies were considered as having a high overall risk of bias [45,75,88].

Except for Matsumura et al. [75], which reported on the insignificant difference in adherence between polypills and multipill therapy and which was assessed as having a high risk of bias, all the other RCTs (*n* = 6, 86%) showed improved adherence when using polypills compared to multipill therapy.

From 60 of the included observational studies, 39 (65%) were assigned eight or nine stars according to the Newcastle–Ottawa rating and were, thus, considered as high-quality studies [5,33,36,37,41,42,43,44,46,47,50,51,53,54,55,56,58,61,62,63,64,65,67,71,72,74,76,78,79,80,81,82,83,84,86,89,90,91,94]. There were 19 studies (32%) that reached criteria for medium quality (six or seven stars) [30,31,32,34,38,39,40,49,57,60,68,69,70,77,85,87,92,93,95], and only two studies (3%) were considered poor quality, with both having five stars assigned [66,73].

**Figure 4:**
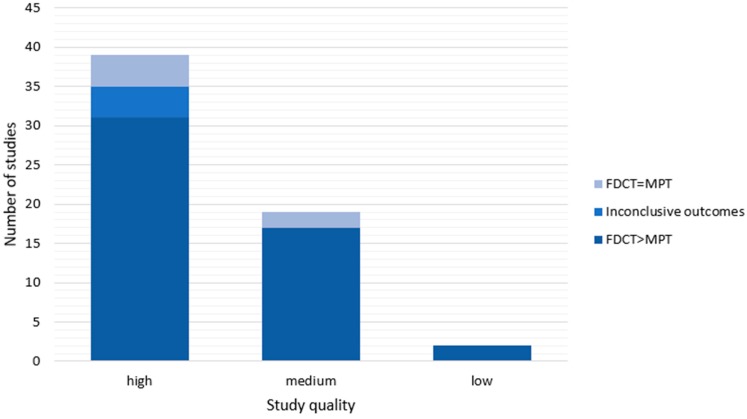
Number of studies with a certain outcome per study quality. FDCT, fixed-dose combination therapy; MPT, multipill therapy. Inconclusive outcomes: see Table 5, Table A2 (Appendix B), and Table A3 (Appendix C) for additional information.

In 50 out of a total of 60 observational studies (83%), adherence to the polypill was shown to be increased compared to multipill therapy. Of the high-quality studies, 31 out of 39 studies (79%) also showed this outcome, which does not differ importantly from findings from the total number of observational studies. Moreover, the ratio of studies with an insignificant difference in adherence to polypill and multipill therapy is very similar for high- and medium-quality studies (4/39 or 10% vs. 2/19 or 11%, respectively). For a visual representation showing the number of studies with a certain outcome concerning adherence per quality of cohort study, see Figure 4.

## 4. Discussion

The main strength of our study is the broad range of included original peer-reviewed studies and that no restrictions concerning the medical condition, type of patients, or adherence measures were used in the research. Based on this systematic review, there is a connection between pill burden and medication adherence in medical conditions such as hypertension, diabetes mellitus type 2, cardiovascular diseases, and HIV. This is reflected by the fact that, in 56 out of 67 examined studies (84%), patient adherence to single-pill fixed-dose combination therapy was significantly higher compared to free-dose combination therapy with multiple pills. Most of the 17 previously conducted meta-analyses and systematic reviews included in our research also suggested a positive effect of polypills on patient adherence. However, four out of 17 studies (24%) did not reach the same conclusion; either the findings were inconclusive [26,29] or FDCT was simply not shown to be superior to multipill therapy [17,23]. It has to be acknowledged, however, that the number of analyzed articles in these studies was either three [17,26], five [23], or six [29]; thus, they might not be highly representative.

Ten percent (*n* = 7) of the individual studies did not observe improved adherence in patients receiving polypill therapy [34,50,58,75,76,90,95]. The authors of these articles suggested the following methodological reasons for their results: (1) the number of participants was too small to obtain significant results [34]; (2) calculation of MPR was made alternatively and therapeutic or in-class switches were allowed for [58]; (3) the pill burden for some multipill therapy regimens was not high enough to have a significant influence on adherence [34]; (4) the study period was not long enough to detect differences between the polypill and multipill groups [75].

Interestingly, two out of 67 studies [61,80], dealing with CVD and HT, respectively, observed both positive and negative outcomes regarding the influence of FDCT on adherence. For the study, dealing with CVD [61], the article’s authors suggested that the reasons for decreased adherence in patients taking polypills were adverse events. These were supposed to be falsely attributed to an active ingredient, which the patients in question were not receiving before the start of the study [61]. Authors of the other study dealing with HT, however, suggested a different reason for decreased adherence [80]. According to them, patients who were highly adherent to their previous treatment with free-combined antihypertensive drugs may not have been taking both of their antihypertensive medications at the same time and as prescribed. Hence, although they were switched to the equivalent FDC, their blood pressure lowered too much; therefore, they reduced the dose of FDCs on their own [80].

To our knowledge, no other previous systematic review in polypills and adherence covered as many original peer-reviewed studies and such a broad range of medical conditions as this. Our findings indicate that the rate at which polypill therapy is associated with higher adherence varies among medical conditions. In most of the studies on CVD, HT, and DMII therapies, adherence increased in patients with polypill therapy; however, in studies on HIV or LUTS therapies, no difference was observed in four out of 15, that is, 27% of the studies. These differences in results can be partly explained by the methodological issues already discussed above. Further research on diseases other than CVD, HT, DMII, and HIV is needed to get a better understanding of whether and how the medical condition influences the impact of reduced pill burden on adherence.

The research mainly revolves around cardiovascular polypills; the reason for this is probably the abundance of patients suffering from CVD and HT [96]. Despite fewer studies on polypills for diabetes and HIV and one for lower urinary tract symptoms associated with benign prostatic hyperplasia, fixed-dose combination therapy was not introduced to other diseases in terms of its potential to influence adherence. As this literature review shows promising results for polypills with 56 out of 67 included studies improving adherence, the research could be extended to a wider range of medical conditions and a wider range of populations and health systems, as well as beyond high-income countries. The current research on polypills is dominated by the research conducted in the USA, reflecting their situation.

There are also some limitations concerning this systematic review. The first one is related to the methodological quality of the selected studies. The results would be more valid if more of the study designs were randomized controlled trials instead of retrospective and prospective cohort studies. Since the study design differed between articles, it was also not possible to assess study quality using only one universal method. Thus, two separate methods were used, one for randomized controlled trials [11] and the other for observational studies [12]. Consequently, it was not possible to make a joint summary of study quality assessment including all articles. Another limitation is related to the countries and the medications included in the studies. Specifically, every country has different public health concerns, as well as health systems, services, and finances, which influence medication practices. Since most of the reviewed studies were from high-income countries, particularly from the USA, a distorted image of the use of polypills in the rest of the world is possible. It must also be acknowledged that patient adherence is affected by many variables, such as patient age, medical condition, and clinical outcomes, which varied significantly in our selected articles. The assessment of these factors was not the aim of this review, but they could have significantly influenced our findings. Furthermore, due to the lack of articles regarding other diseases, it is not possible to conclude whether polypills are associated with an increase in medication adherence on a general level. This can become clear only when more studies regarding the effect of polypills on adherence in other diseases are conducted. Moreover, there is some overlapping among previously published systematic reviews and meta-analyses, as some articles were included in more than one of them, thereby giving those studies more emphasis.

Another issue that must be acknowledged as a possible limitation to our study is the diversity of methods for measuring adherence that were applied in the included studies. In 67 articles, 11 different adherence measures were used, which makes the results of the studies more difficult to compare, thereby adding a possible source of bias. The methods range, on the one hand, from assessing prescription claims and the number of the pills taken (indirect methods) to analyzing questionnaires and patient self-assessment, on the other hand (subjective methods) [75], all having specific advantages and limitations. For example, indirect methods are a very technical way of measuring adherence, but easy to apply to bigger datasets, which may explain their use in these studies. However, unlike self-assessment-based methods, they do not cover other aspects of a patient´s life that also influence adherence. Additionally, pill-count and prescription claim analyses do not guarantee that the patients were taking the prescribed medicines [40,49]. On the other hand, self-assessment is a very subjective way of measuring adherence and is often prone to over-reporting the actual state [45,87]. In none of the studies were direct methods of measuring adherence used (i.e., measuring blood concentration of the active ingredients), which are the most objective methods, but also the most difficult, time-consuming, and expensive, as well as being inconvenient for patients [48]. In the future, it might be useful to consider the aspect of how patients perceive taking one instead of multiple pills in the methods for assessing adherence. Finally, due to such substantial heterogeneity between studies in terms of study design and reporting on the outcome, a pooled estimate of the effect of the pill burden on adherence was not analyzed, which can be considered as one of the limitations as well.

Another issue that emerged while carrying out this systematic review relates to the vague use of the terms “adherence” and “persistence.” Certain methods (e.g., duration to treatment discontinuation) were defined as a measure of adherence in some of the studies, whereas the other studies stated it as a method of assessing therapy persistence. In the future, clearer definitions and distinction between these two terms and the methods used for measuring them should be made available to avoid misconceptions about the aims of the studies.

The years of publication of selected articles indicate that there was increased interest in polypills in the last years. Only seven of the selected articles (10%) were published before 2008, and 35 out of a total of 67 studies (52%) were published in the last six years. One of the reasons for this rising trend might be the fact that fixed-dose combination therapy shows promising results for improving patient adherence.

Most of today’s commercially available polypills are intended for the treatment of only one indication. However, since it was already established how beneficial FDCT can be for patients, another interesting concept that is not yet applied very often, but is worth considering, is combining drugs for different indications into one formulation. Between 2010 and 2015, two FDCs composed of active pharmaceutical ingredients (APIs) for different comorbid diseases were already approved, both without full clinical study data [97]. In the future, more emphasis could be given to such FDCs, since that would reduce pill burden even more and, hopefully, have an even greater effect on patient adherence.

Even though this systematic review shows one of the potential benefits of polypill therapy, some of its disadvantages should also be acknowledged. For example, if dosing titration is needed, fixed-dose combination therapy can be inflexible if the appropriate dosage is not available in the form of a polypill [98,99,100,101]. That could lead to exposure of patients to unnecessary therapy and even adverse effects without added benefits [100]. Furthermore, if adverse effects occur, it cannot be possible for the patient to determine which of the components is causing them [101]. Another possible issue is that polypill therapy may be more expensive than multipill therapy [66,67,98].

All in all, the evidence shown in this systematic review constitutes a base for possible advantages of polypill therapy over multipill therapy, at least in the investigated medical conditions, when tackling the widespread and alarming problem of patient adherence to medication. Thus, the role of polypills in clinical practice should not be neglected, even though their contribution to increasing adherence is only partial. There are many other patient- and system-related factors, such as patient age and socioeconomic status, health literacy, disease and medication beliefs, adverse effects, medical condition and its seriousness, treatment costs, and clinical outcomes, which also play a major role in achieving positive outcomes [48,52,61,80,84]. However, reducing the complexity of pill regimens, especially in diseases where the number of pills can seem overwhelming for patients, could at least partly lead to increased medication adherence and, therefore, also improved clinical outcomes. Nonetheless, to better understand the role of polypills in clinical practice, a higher number of long-term randomized controlled trials dealing with different medical conditions will be needed.

## 5. Conclusions

This systematic review shows a connection between pill burden and medication adherence. In most of the included studies, adherence to polypill therapy was significantly higher compared to multipill therapy. Our findings indicate that the rate at which fixed-dose combination therapy is associated with higher adherence varies between medical conditions. As this systematic review shows promising results for polypills, research could be extended to a wider range of medical conditions, populations, and health systems, as well as beyond high-income countries.

## Figures and Tables

**Table 1:** Summary of conclusions per disease in previously published systematic reviews and meta-analyses (*n* = 17).

Disease	Conclusions Concerning Adherence to FDCT	Study Design [Reference]		
		Meta-Analysis	Systematic Review with Meta-Analysis	Systematic Review
Various diseases (*n* = 2)	FDCT > MPT (*n* = 1)	[13]		
	Inconclusive (*n* = 1)		[29]	
Hypertension (*n* = 5)	FDCT > MPT (*n* = 4)	[14,15,16]	[25]	
	Inconclusive (*n* = 1)		[26]	
CVD (*n* = 3)	FDCT > MPT (*n* = 2)	[18]		[21]
	FDCT = MPT (*n* = 1)	[17]		
HIV (*n* = 4)	FDCT > MPT (*n* = 4)	[19,20]	[24,27]	
Diabetes (*n* = 2)	FDCT > MPT (*n* = 2)		[28]	[22]
Tuberculosis (*n* = 1)	FDCT not favored (*n* = 1)		[23]	

FDCT, fixed-dose combination therapy; MPT, multipill therapy; CVD, cardiovascular disease; HIV, human immunodeficiency virus.

**Table 2:** General information about reviewed articles (*n* = 67).

Information of Interest	Result (Number of Studies with a Certain Feature)	References
**Disease**	CVD (*n* = 11)	[35,45,48,49,52,58,59,60,61,62,63]
	HT (*n* = 31)	[5,33,38,39,40,41,42,46,53,55,56,64,65,66,67,68,69,70,71,72,73,74,75,76,77,78,79,80,81,82,83]
	DMII (*n* = 10)	[31,32,36,43,44,51,54,84,85,86]
	HIV (*n* = 14)	[30,34,37,47,50,57,87,88,89,90,91,92,93,94]
	LUTS/BHP (*n* = 1)	[95]
**Country where the study was conducted (in alphabetical order)**	Argentina (*n* = 1)	[35]
	Australia (*n* = 3)	[45,58,61]
	Austria (*n* = 1)	[66]
	Belgium (*n* = 2)	[66,88]
	France (*n* = 1)	[59]
	Germany (*n* = 4)	[7,66,67,79]
	Greece (*n* = 1)	[85]
	India (*n* = 1)	[52]
	Ireland (*n* = 1)	[52]
	Italy (*n* = 8)	[35,40,42,69,70,84,87,92]
	Japan (*n* = 2)	[73,75]
	Korea (*n* = 1)	[64]
	The Netherlands (*n* = 5)	[52,59,66,88,95]
	New Zealand (*n* = 1)	[48]
	Paraguay (*n* = 1)	[35]
	Romania (*n* = 1)	[77]
	Spain (*n* = 2)	[35,90]
	Switzerland (*n* = 1)	[66]
	Taiwan (*n* = 5)	[5,53,71,78,80]
	UK (*n* = 1)	[52]
	USA (*n* = 36)	[30,31,32,33,34,36,37,38,39,41,43,44,46,49,50,51,54,55,56,57,60,62,63,65,68,72,74,76,81,82,83,86,89,91,93,94]
**Follow-up period (given as the exact, average, or minimal value, depending on the study)**	6 weeks (*n* = 1)	[91]
	2 months (*n* = 4)	[37,57,89,93]
	3 months (*n* = 1)	[73]
	18 weeks (*n* = 1)	[59]
	24 weeks (*n* = 1)	[85]
	6 months (*n* = 16)	[30,32,42,43,44,46,51,54,55,58,66,68,70,72,75,87]
	9 months (*n* = 1)	[35]
	12 months (*n* = 26)	[31,33,36,38,39,40,41,48,49,52,56,60,62,64,65,67,71,74,76,80,81,82,83,84,86,95]
	15 months (*n* = 1)	[78]
	18 months (*n* = 3)	[34,45,50]
	1.7 years (*n* = 1)	[94]
	96 weeks (*n* = 1)	[90]
	24 months (*n* = 5)	[5,53,61,69,88]
	33 months (*n* = 1)	[47]
	36 months (*n* = 1)	[63]
	4 years (*n* = 1)	[77]
	5 years (*n* = 2)	[79,92]
**Year of publication**	2002–2004 (*n* = 3)	[32,43,49]
	2005–2007 (*n* = 4)	[40,54,60,89]
	2008–2010 (*n* = 15)	[30,33,36,38,39,41,44,46,51,55,56,62,68,72,87]
	2011–2013 (*n* = 10)	[31,34,37,52,63,65,73,75,83,84]
	2014–2016 (*n* = 22)	[5,35,42,45,47,48,50,57,58,59,66,70,76,78,80,81,82,85,86,88,91,93]
	2017–2019 (*n* = 13)	[14,53,61,64,69,71,74,77,79,90,92,94,95]
**Study design**	Randomized clinical study (*n* = 7)	[35,45,48,52,59,75,88]
	Retrospective cohort study (*n* = 52)	[5,31,32,33,36,37,38,39,40,41,42,43,44,46,47,49,50,51,53,54,55,56,57,58,60,61,62,63,64,65,67,68,69,70,71,72,74,76,77,78,79,80,81,82,83,84,86,89,92,93,94,95]
	Prospective cohort study (*n* = 8)	[30,34,66,73,85,87,90,91]
**Number of drugs in the polypill examined in the study**	Two drugs (*n* = 41)	[5,31,32,33,36,38,39,40,41,42,43,44,46,49,51,53,54,55,56,58,60,61,62,63,64,65,67,68,69,70,71,72,73,75,78,79,80,83,85,89,95]
	Three drugs (*n* = 11)	[30,34,35,37,47,50,66,81,82,87,88]
	Four drugs (*n* = 5)	[45,48,52,59,90]
	Five drugs (*n* = 1)	[57]
	Not mentioned (*n* = 9)	[74,76,77,84,86,91,92,93,94]

CVD, cardiovascular disease; DMII, diabetes mellitus type 2; LUTS/BHP, lower urinary tract symptoms associated with benign prostatic hyperplasia; HT, hypertension; HIV, human immunodeficiency virus; UK, United Kingdom; USA, United States of America.

**Table 3:** Visualization of number of active ingredients contained in a polypill. Written in the table are numbers of the studies with the given characteristics (disease and number of active ingredients in the polypill).

Disease	Number of Studies Dealing with a Polypill Containing a Given Number of Active Ingredients (2, 3, 4, 5, or Not Mentioned)				
	II	III	IV	V	Not mentioned
CVD	6 [49,58,60,61,62,63]	1 [35]	4 [45,48,52,59]	0	0
HT	25 [5,33,38,39,40,41,42,46,53,56,64,65,67,68,69,70,71,72,73,75,78,79,80,83,94]	3 [66,81,82]	0	0	3 [74,76,77]
DMII	8 [31,32,36,43,44,51,54,85]	0	0	0	2 [84,86]
HIV	1 [89]	7 [30,34,37,47,50,87,88]	1 [90]	1 [57]	4 [91,92,93,94]
LUTS/BPH	1 [95]	0	0		0
Sum	41	11	5	1	9

CVD, cardiovascular disease; DMII, diabetes mellitus type 2; LUTS/BHP, lower urinary tract symptoms associated with benign prostatic hyperplasia; HT, hypertension; HIV, human immunodeficiency virus.

**Table 4:** Methods for measuring adherence applied in the articles (*n* = 67).

Method	Study-Specific/General	Short Description	Assessment of Level of Adherence	*n* of Studies [References]
Medication possession ratio (MPR)	General	Uses pharmacy prescription claims calculated as the number of days’ supply divided by the number of days between the first refill and the end of the follow-up period	Low adherence: MPR < 0.5 Intermediate adherence: MPR = 0.5–0.8 High adherence: MPR > 0.8	*n* = 30 [5,31,32,33,36,39,40,41,43,44,49,50,51,54,55,57,58,60,62,63,64,67,80,82,84,86,89,91,94,95]
Proportion of days covered (PDC)	General	Uses prescription claims data; every day has to be covered by the medication; coverage is calculated based on the refill data. For example, if the patient has 30 pills in his prescription (1/day) and he gets a refill after 40 days, his PDC is 30/40 or 75%.	A PDC of >80% is considered adherent.	*n* = 21 [38,42,46,53,56,61,65,68,69,70,71,72,74,76,78,79,81,82,83,90,93]
Pill count	General	Healthcare professional pays an unexpected visit to the patient’s home and counts the pills left; difference between the number of pills dispensed and the number of pills not taken gets divided by number of prescribed pills.	Patient is considered adherent, if the percentage is between 80% and 110%.	*n* = 6 [30,35,47,73,75,91]
Morisky scale	General	Questionnaire containing eight questions; a self-assessment scale.	Based on the sum of the scores.	*n* = 3 [35,59,66]
Self-reporting	Study-specific	1. Asking the patients about the names and dosages of all drugs that are currently taken [48]. 2. Self-reported use of indicated combination treatment (antiplatelet, statin, and ≥2 blood-pressure-lowering therapies for ≥4 of the previous seven days) [45,52]. 3. Self-reporting of missed doses at each medical visit [50]. 4. Completing a compliance questionnaire—nine questions about the names and dosages of all drugs, missing doses, treatment interruptions, etc. [85]	1. Adherent: patients reported taking an antiplatelet, statin, and two or more blood-pressure-lowering drugs. Non-adherent: patients who forgot one or more drugs. 2. Level of adherence not assessed. 3. It was assumed that each day of ART missed was an additional day between refills of a 30-day supply → MPR method was applied. 4. Adherent: not missing any drug dose or no more than 2 doses per week, received the correct dosage of the medication, and not interrupting their treatment.	*n* = 5 [45,48,50,52,85]
Visual Analog Scale (VAS)	General	Uses information given by the patient who performs self-assessment of adherence on a scale 0–100.	Non-adherent: 0 Perfectly adherent: 100	*n* = 2 [34,87]
Simplified Medication Adherence Questionnaire (SMAQ)	General	Self-reported questionnaire focused on HIV patients, containing six items.	Method of assessment is not given in the article.	*n* = 1 [88]
Prescription records review	Study-specific	Computing the total number of consecutive months that was covered by antihypertensive prescriptions during the study; adherence is expressed as percentage of time.	Low adherence: <20% Medium adherence: 20–79% High adherence: ≥80%	*n* = 1 [77]
Electric adherence monitoring	General principle, study-specific design (depends on the dosage form, dosage regimen, etc.)	The medication vial was closed with a cap containing a microprocessor, which was recording date and time of all openings. The vial was filled with the exact amount of medication required for the complete treatment period. The participant was instructed not to open the vial except when taking the medication according to the prescribed regimen.	Based on whether the patient was taking the doses daily and according to the schedule.	*n* = 1 [59]
Time to the first instance to discontinuation *	General method, study-specific definition	Defined as no repeat of prescription within 150% of the previous days’ supply.	Treatment discontinuation: break of therapy for more than 150% of the previous days’ supply.	*n* = 1 [79]
RDD/PDD ratio	General	Ratio between received daily dose (corresponds to the ratio between total doses received and treatment days) and prescribed daily dose (stands for the intention to treat and the real prescriptive tendency).	Adherence is assessed and given only as an RDD/PDD ratio; there is no evaluation of what is considered high or low adherence.	*n* = 1 [92]

* Usually used as a measure of therapy persistence. ART, antiretroviral therapy; RDD, received daily dose; PDD, prescribed daily dose.

**Table 5:** Summary of the study results per disease. Statistically significant differences in adherence outcomes are presented and considered.

Disease	Comparison of Adherence Outcome between FDCT and MPT; Number of Studies with Certain Result Is Given in Parenthesis	References
CVD (*n* = 11)	FDCT > MPT (*n* = 9)	[35,45,48,49,52,59,60,62,63]
	FDCT = MPT (*n* = 1)	[58]
	Inconclusive * (*n* = 1)	[61]
HT (*n* = 31)	FDCT > MPT (*n* = 28)	[5,33,38,39,40,41,42,46,53,55,56,64,65,66,67,68,69,70,71,72,73,74,77,78,79,81,82,83]
	FDCT = MPT (*n* = 2)	[75,76]
	Inconclusive * (*n* = 1)	[80]
DMII (*n* = 10)	FDCT > MPT (*n* = 9)	[31,32,36,44,51,54,84,85,86]
	Inconclusive * (*n* = 1)	[43]
HIV (*n* = 14)	FDCT > MPT (*n* = 10)	[30,37,57,87,88,89,91,92,93,94]
	FDCT = MPT (*n* = 3)	[34,50,90]
	Inconclusive * (*n* = 1)	[47]
Other (*n* = 1)	FDCT = MPT (*n* = 1)	[95]

* Several outcomes were observed (FDCT < MPT or FDCT > MPT or FDCT = MPT). See Table A2 (Appendix B) and Table A3 (Appendix C) for additional information. FDCT, fixed-dose combination therapy; MPT, multipill therapy; CVD, cardiovascular disease; DMII, diabetes mellitus type 2; LUTS/BHP, Lower urinary tract symptoms associated with benign prostatic hyperplasia; HIV, human immunodeficiency virus.

## Appendix A

**Table A1:** Summary of the systematic reviews and meta-analyses (*n* = 17) regarding the medical condition in focus, study aim, number and design of included studies, and main results.

Author, Reference, Year of Publication, Study Design	Medical Condition or Disease in Focus	Study Aim	Number and Design of Studies Included	Main Results
Various diseases (*n* = 1)				
Van Galen et al. [29], 2014 Systematic review and meta-analysis	HIV (*n* = 2), tuberculosis (*n* = 3), hypertension (*n* = 1)	To summarize and synthesize existing evidence from RCTs about the effect on adherence to FDCT versus the same drugs administered as separate pills	6 RCTs	Administering drugs as FDC increased the likelihood of optimal adherence (OR 1.33 (95% CI, 1.03–1.71)); however, the difference was statistically significant only for HIV. Other diseases only showed the same trend.
Bangalore et al. [13], 2007 Meta-analysis	Tuberculosis (*n* = 2), hypertension (*n* = 4), DMII (*n* = 2), HIV (*n* = 1)	To evaluate the effect of FDCT on patient adherence to medication	9: 3 RCTs, 6 retrospective database analyses	Utilizing FDC resulted in 26% decrease in the risk of non-compliance compared to the free-drug therapy (RR: 0.74; 95% CI: 0.69–0.80; *p* < 0.0001).
Hypertension (*n* = 5)				
Kawalec et al. [25], 2018 Systematic review with meta-analysis and narrative synthesis	Hypertension	To present an up-to-date evaluation of the effectiveness of FDCs and free equivalent combinations in management of hypertension and to get more accurate results by using a stratified meta-analysis	Whole systematic review: 26 clinical studies, 2 systematic reviews Meta-analysis: 12; 11 retrospective cohort studies, 1 nonrandomized trial (assessing adherence)	FDC were shown to be associated with an improvement in adherence in comparison to free equivalent combination therapy; e.g., meta-analysis of 4 cohort studies showed an increased adherence with FDCT in the average MPR by 13.1% (95% CIs: 8.9%–17.2%, *p* < 0.001).
Du et al. [14], 2018 Meta-analysis	Hypertension	To assess the effect of FDCT on medication adherence in comparison to free-equivalent combination therapies in management of hypertension	7 (assessing adherence): 6 retrospective studies, 1 prospective study	FDCT was associated with higher medication adherence than free equivalent combination therapies; mean difference was 14.92% (95% CIs: 7.38%–22.46%).
Sherrill et al. [15], 2011 Meta-analysis	Hypertension	To compare healthcare resource use costs, adherence, and persistence between groups of patients on single-pill and free-equivalent combination therapies	7 retrospective studies (assessing adherence)	The average MPR was 8% higher in the patient group to prior antihypertensives and 14% higher in experienced FDCT patient group, compared with corresponding free-equivalent combination group.
Gupta et al. [16], 2009 Meta-analysis	Hypertension	To compare compliance, persistence, blood pressure control, and safety between FDCTs and free-drug combinations	5 (assessing adherence): 2 RCTs, 3 retrospective cohort studies	The use of FDCT was associated with significantly better compliance (OR: 1.21, 95% CIs: 1.03–1.43; *p* = 0.02).
Mallat et al. [26], 2016 Systematic review and meta-analysis	Essential arterial hypertension	To compare the effects of FDCT and free combination therapy with blood pressure lowering agents in the management of essential hypertension	3 RCTs (assessing adherence)	Two articles reported no difference in adherence between groups, one article showed increased adherence in FDCT group.
CVD (*n* = 2)				
Selak et al. [17], 2018 Meta-analysis	CVD	To assess the impact of FDCT on achieving the 2016 European Society of Cardiology guideline targets for blood pressure, low-density lipoprotein, cholesterol, and antiplatelet therapy	3 RCTs	No difference was observed between groups in antiplatelet adherence (96% vs. 96%, RR: 1.00, 95% CIs: 0.98–1.01).
Bahiru et al. [21], 2017 Systematic review	Atherosclerotic CVD	To study the effect of FDC therapy on all-cause mortality, fatal and non-fatal ASCVD events, adverse events, blood pressure, lipids, adherence, discontinuation rates, health-related quality of life and costs	4 RCTs (assessing adherence)	FDC therapy improved adherence by 44% (26% to 65%) compared with usual care.
Webster et al. [18], 2016 Meta-analysis	CVD	To compare FDCT with usual care in patients with CVD or at high risk	3 RCTs	Participants in the FDC group had higher adherence than patients with usual care (80% vs. 50%, RR: 1.58; 95% CIs: 1.32–1.90; *p* < 0.001).
Diabetes (*n* = 2)				
Han et al. [28], 2012 Systematic review and meta-analysis	DMII	To compare effects of FDCs and dual therapy of antihyperglycemic agents on glycemic control and adherence	8 cohort studies (assessing adherence)	Five comparisons FDC versus dual therapy cohorts showed significantly higher MPR with FDC (MD = 8.6% (95% CIs: 1.6–15.6); *p* = 0.0162). Three comparisons showed results for patients who switched from dual therapy to FDC or stayed on dual therapy, with higher MPR for patients who switched to FDC (MD = 5.0% (95% CIs: 3.1–6.8); *p* < 0.0001).
Hutchins et al. [22], 2011 Systematic review	DMII	To evaluate adherence, patient-reported outcomes, costs, resource use and cost effectiveness between FDCT and LDCT	8 cohort studies (assessing adherence)	Adherence was improved with using FDCT instead of LDCT.
HIV (*n* = 4)				
Altice et al. [27], 2019 Systematic review and meta-analysis	HIV	To study the relationship between single or multiple tablet regimens and treatment adherence and viral suppression	Whole systematic review: 11 prospective or retrospective non-randomized studies (assessing adherence); 10 full texts and one conference abstract Meta-analysis: 8; 7 full texts and one conference abstract	Polypills were associated with higher treatment adherence than multipill therapy in 10 studies: a 63% greater likelihood of achieving ≥95% adherence (95% CIs: 1.52–1.74; *p* < 0.001) and a 43% increase in the likelihood of achieving ≥90% adherence (95% CIs: 1.21–1.69; *p* < 0.001).
Clay et al. [24], 2018 Systematic review, meta-analysis	HIV	To compare single-pill to multi-tablet regimens in HIV treatment by using published data	Reporting on adherence: 30, but only 8 observational studies reported quantifiable data and were included in the meta-analysis.	Patients utilizing single-pill regimens were significantly more adherent (OR: 1.96, *p* < 0.001).
Clay et al. [20], 2015 Meta-analysis	HIV	To compare patient adherence and clinical and economic outcomes of FDCT and multipill therapy regimens	Reporting on adherence: 20; but only 5 having quantifiable or analyzable data for meta-analysis: 4 observational studies, 1 economic models-based study.	Patients on FDCT were more adherent than patients on multipill therapy regimen of any frequency (OR: 2.37, 95% CIs: 1.68–3.35; *p* < 0.001; 4 studies).
Ramjan et al. [19], 2014 Meta-analysis	HIV	To compare the advantages of FDC antiretroviral therapy to separate pill therapy regimens for patients and programs	Reporting on adherence: 10, but only 7 included in the quantitative analysis: 5 RCTs and 2 retrospective cohort studies.	RCTs showed better adherence in FDCT group than in separate pill regimens (RR: 1.10, 95% CIs: 0.98–1.22); observational studies showed the same trend (RR: 1.17, 95% CIs: 1.07–1.28).
Tuberculosis (*n* = 1)				
Albanna et al. [23], 2013 Systematic review and meta-analysis	Tuberculosis	To assess different aspects of management of tuberculosis using FDC or free combination treatment	5 RCTs (assessing adherence)	None of the studies favored FDCT.

FDCT, fixed-dose combination therapy; FDC, fixed-dose combination; RCT, randomized controlled trial; MPR, medication possession ratio; MD, mean difference; CVD, cardiovascular disease; ASCVD, atherosclerotic CVD; HIV, human immunodeficiency virus; DMII, diabetes mellitus type II; CI, confidence interval; OR, odds ratio; RR, relative risk.

## Appendix B

**Table A2:** Summary of the original articles (*n* = 67) regarding aim, study setting, follow-up period, population, type of adherence outcome measures, and results.

Author, Reference, Year of Publication, Study Country, Study Design	Study Aim, Study Setting and Follow-Up Period	Study Population	Outcome Measures	Main Results (Concerning Adherence)
CVD (*n* = 11)				
Castellano et al. [35], 2014, Argentina, Paraguay, Italy and Spain Phase 1: observational, prospective, cross-sectional study Phase 2: randomized, controlled clinical trial	Phase 1: to identify factors interfering with adherence to CV medications for secondary prevention after an acute myocardial infarction. Phase 2: to test the impact of a polypill on adherence, blood pressure, low-density lipoprotein cholesterol, safety and tolerability. Phase 1: 64 outpatient clinics in Argentina, Brazil, Paraguay, Italy and Spain Phase 2: 63 clinics in Argentina, Paraguay, Italy and Spain Follow-up period: 9 months	Phase 2: 695 infarct patients ≥40 years of age with a history of acute myocardial infarction within the last 2 years (350 on FDC therapy and 345 on conventional multipill treatment).	Adherence was measured via Morisky Medication Adherence Scale and pill count.	Polypills showed a significantly higher adherence in comparison with multiple pills (50.8% vs. 41%, *p* = 0.019).
Lafeber et al. [59], 2014, the Netherlands Randomized controlled trial	To compare the morning and evening administration of a cardiovascular polypill and to assess the effect of the polypill on patients’ clinical outcomes, adherence, and preference compared to the separately administered identically dosed drugs University Medical Center Utrecht Follow-up period: 18 weeks	78 patients with established atherosclerotic CVD and an indication for the use of cardiovascular medication (during the three treatment periods of 6 weeks, each was receiving every type of therapy regimen (polypill in the morning, polypill in the evening, and mutlipill therapy with individual drugs), but in different sequences).	Adherence was measured via microelectronic monitoring device and Morisky Medication Adherence Scale.	According to digital adherence monitoring, adherence was 5.2% (95% CIs: 1.4%–9.1%) higher when using the polypill in the morning and 5.3% (95% CIs: 1.4%–9.1%) higher when using the polypill in the evening compared to multipill therapy. Morisky scale recognized non-adherence in 4 (5%) participants when using the polypill in the morning, in 6 (8%) participants when using the polypill in the evening, and in 10 (13%) participants when using the individual agents (*p* = 0.22).
Patel et al. [45], 2014, Australia Randomized controlled trial	To determine if polypills improve adherence in high risk CVD patients 33 Australian health centers Follow-up period: 18 months	623 patients ≥18 years of age with high CVD risk (311 allocated to polypill treatment and 312 to conventional treatment).	Adherence was measured via self-reporting.	Patients on the polypill therapy reported an adherence rate of 70.1% at study end, while people on usual care reported a 46.9% adherence (*p* < 0.001).
Selak et al. [48], 2014, New Zealand Randomized controlled trial	To investigate the impact of FDCT on the adherence rate and risk factor control in patients with high cardiovascular risk 54 general practices all over New Zealand Follow-up period: 12 months	513 patients aged 18–79 years at high risk of CVD (256 allocated to FDC and 257 to usual care).	Adherence was measured via self-reporting.	Adherence in patients receiving FDCT was higher compared to the two-pill treatment (81% vs. 46%, *p* < 0.001).
Thom et al. [52], 2013, UK, India, Ireland, the Netherlands Randomized, open-label, blinded-end-point clinical trial	To assess the impact of a polypill in comparison to usual care on adherence patterns, systolic blood pressure and low-density lipoprotein cholesterol Patient data obtained via databases, hospitals and general practices in India, England, Ireland, and the Netherlands Follow-up period: 12 months	2004 patients ≥18 years of age with high cardiovascular risk, defined as either established CVD, or an estimated 5-year CVD risk of 15% or greater (1002 allocated to FDC group and 1002 to usual care)	Adherence was measured via self-reporting.	The FDCT group had significantly improved adherence compared to the usual care group (88% vs. 65%, *p* < 0.001).
Schaffer et al. [61], 2017, Australia Retrospective cohort study	To compare adherence in patients initiating amlodipine/atorvastatin therapy as an FDC or free combination and to identify subgroups benefiting most from FDCs Data retrieved via Australian Pharmaceutical Benefits Scheme Follow-up period: 24 months	9430 patients, who started their therapy with study drugs either as an FDC or in free combination (3996 on FDC and 5434 on free combination therapy).	Adherence was measured via PDC.	Patients initiating on an FDC were more likely to have near-perfect adherence compared to those with the free combination, if they were previously statin adherent irrespective of amlodipine dose (amlodipine 5 mg: OR = 1.61, 95% CIs: 1.38–1.87; amlodipine 10 mg: OR = 2.39, 95% CIs: 1.63–3.51), or if they were previously statin nonadherent and initiated on the FDC with 5-mg amlodipine (OR = 1.87, 95% CIs: 1.50–2.32). However, statin-naïve initiating on FDCT with 10-mg amlodipine were less likely to have near-perfect adherence (OR = 0.60, 95% CIs: 0.41–0.88) and more likely to have early nonadherence (OR = 1.73, 95% CIs: 1.17–2.55) compared with the free combination.
Bartlett et al. [58], 2016, Australia Retrospective cohort study	To compare adherence and persistence in patients who add ezetimibe to statin therapy as a separate pill combination or FDC Data retrieved via Australian Pharmaceutical Benefits Scheme Follow-up period: 6 months	9391 patients, who initiated ezetimibe as separate pill or ezetimibe in FDC (3651 on multipill therapy and 5740 on FDC therapy).	Adherence was measured via MPR.	Adherence was similar in both groups; mean MPRs: multipill therapy = 0.99 (95% CIs: 0.98–1.01) and FDC = 0.97 (95% CIs: 0.95–0.99).
Kamat et al. [63], 2011, USA Retrospective cohort study	To compare adherence between single- and multipill therapies with lipid-modifying drugs Data retrieved via HealthCore Integrated Research Database Follow-up period: 36 months	42,460 patients ≥18 years of age newly initiating FDC dyslipidemia therapy (38,847 patients) or equivalent multipill therapy (3613 patients).	Adherence was measured via MPR.	The mean PDC was 0.76 (±0.26) and 0.70 (±0.27) in the first 3 months of treatment, 0.54 (±0.40) and 0.45 (±0.40) in the second 3 months of treatment, and 0.50 (±0.41) and 0.41 (±0.43) for the remaining 30 months for FDC and multipill groups, respectively. Average PDC was significantly higher in the SPC group (0.56 ± 0.34) than in the LDC group (0.47 ± 0.33), *p* < 0.0001.
Balu et al. [62], 2009, USA Retrospective cohort study	To compare adherence between patients treated with the FDC multipill combination therapy, to assess the relationship between optimal adherence and CVD-associated total healthcare resource utilization and healthcare cost Data retrieved via HealthCore Integrated Research Database Follow-up period: 12 months	8988 patients ≥18 years of age newly initiating FDC (niacin extended-release (NER) and lovastatin (NERL); 6638 patients) or multipill combination therapies (NER and simvastatin (NER/S); 1687 patients, or lovastatin (NER/L); 663 patients) between index dates.	Adherence was assessed via MPR.	NER/S and NER/L patients were 31.3% (95% CIs: 22.9%–39.5%) and 39.1% (95% CIs: 26.7%–49.4%) less likely to be adherent than NERL patients (*p* < 0.01).
LaFleur et al. [60], 2006, USA Retrospective cohort study	To compare patient adherence between different pill regimen of lipid-lowering drugs Patient data retrieved from RxAmerica database Follow-up period: mean ca. 12 months	1672 patients who started the therapy with any of the study drugs in the selection years (among them, 224 in the ERNL (= polypill) group and 347 in the ERN-S (= combination therapy) group.	Adherence was measured via MPR.	Adherence rates for ERNL (= polypill) and ERN-S (two pills) groups were significantly different: 72.5% vs. 75.8% (*p* = 0.033).
Taylor and Shoheiber [49], 2003, USA Retrospective cohort study	To check if adherence is better for a single-pill regimen vs. a multiple-pill regimen. Patient data retrieved from a managed care organization that provides benefits for members enrolled in various health plans Follow-up period: 12 months	5732 patients aged 18–64 years with a diagnosis code for HT and who were treated with one of the two study regimens and filled at least two prescriptions for their regimen on two different dates during the study period (2754 receiving FDC and 2978 receiving multipill therapy).	Adherence was measured via MPR.	The overall adherence rate in the polypill group (80.8%) was significantly higher than in the multipill group (73.8%), *p* < 0.001.
Hypertension (*n* = 31)				
Matsumara et al. [75], 2012, Japan Randomized controlled trial	To investigate if medication adherence in hypertensive patients would improve with SPC 29 hospitals or clinics in Japan Follow-up period: 6 months	207 hypertensive patients ≥20 years of age (103 allocated to FDC therapy and 104 to multipill therapy).	Adherence was measured via residual pill count.	No significant differences were found in adherence rate between SPC and multiple-pill groups (*p* = 0.89).
Bramlage et al. [66], 2014, Austria, Belgium, Germany, the Netherlands, and Switzerland Prospective, non-interventional multicenter study	To get information on safety, tolerability and efficacy of the FDC of olmesartan/amlodipine/hydrochlorothiazide in daily practice and to check the impact of polypills on adherence in patients with HT Primary care practice in five European countries (Austria, Belgium, Germany, the Netherlands, and Switzerland) Follow-up period: 6 months	14,979 patients ≥18 years of age with essential HT and new treatment with an FDC.	Adherence was measured via a Morisky Medication Adherence Scale.	Mean adherence raised from 6.0% to 6.9% when switching from multipill to FDCT (*p* < 0.001).
Kumagai et al. [73], 2012, Japan Prospective, multicenter, observational study	To investigate the impact of FDC treatment on adherence, blood pressure and healthcare costs Several clinics and hospitals in Japan Follow-up period: 3 months	196 patients with hypertension treated with free-drug combinations of ARB and amlodipine; free-drug combinations were replaced with the same dose of the FDC.	Adherence was measured via self-reported pill-count.	Adherence was significantly improved after switching from free combination to FDC therapy (*p* < 0.01).
Ah et al. [64], 2019, Korea Retrospective cohort study	To compare adherence and persistence between single-pill and free equivalent combination and between two single-pill combinations as initial treatment hypertensive patients who also received prepackaged medications from the pharmacy Data retrieved via Korean national claims database Follow-up period: 12 months	40,350 patients ≥18 years of age with ICD-10 code of hypertension and started on combination regimen consisting of an ARB and either a thiazide diuretic or CCB (20,175 on multipill therapy and 20,175 on single-pill therapy).	Adherence was measured via MPR.	The single-pill cohort had 30% higher medication adherence (OR 1.31, 95% CIs: 1.25–1.37) than the free pill cohort (*p* < 0.05).
Bramlage et al. [67], 2018, Germany Retrospective cohort study	To assess the effect of FDCs on persistence, adherence, and medication costs, to acquire data regarding the differences in patient characteristics and comedications between patients prescribed an FDC and those prescribed a free-dose combination, and to assess motivations behind prescription of one or another of the combination therapy types Data retrieved via IMS® Disease Analyzer, which contains medical records provided by 2500 physician practices in Germany Follow-up period: 12 months	81,958 hypertensive patients who filled at least one prescription for one of two drugs combinations, either as a single-pill FDC or as a two-pill free-dose combination (10,938 on ramipril/amlodipine FDCT, 60,525 on ramipril/amlodipine free dose therapy, 1413 on candesartan/amlodipine FDCT, 9082 on candesartan/amlodipine free dose therapy).	Adherence was assessed via MPR.	The mean MPR was higher for patients prescribed FDC compared to those taking a free-dose combination (ramipril/amlodipine: 0.72 vs. 0.58, *p* < 0.001; candesartan/amlodipine: 0.92 vs. 0.79, *p* < 0.001).
Degli Esposti et al. [69], 2018, Italy Retrospective cohort study	To assess the changes in treatment adherence in patients who switched from single-pill or two-pill therapy to FDCT Data retrieved via administrative databases involving three local health units in three Italian regions Follow-up period: 24 months	24,020 patients ≥18 years of age receiving at least one prescription of selected antihypertensive drugs in selection period (1093 with two-pill treatment, 302 switched to FDCT, 791 did not; 22,927 with MT, 3295 switched to FDCT, 19,632 did not).	Adherence was measured via PDC.	Adherence rose significantly among the subjects who switched to FDC from two-pill therapy (+13%, *p* < 0.001), while it was almost unchanged or slightly decreased among the subjects who did not (−4%, *p* < 0.001).
Ho et al. [71], 2018, Taiwan Retrospective cohort study	To compare the clinical outcomes of FDC vs. free combinations of renin–angiotensin system inhibitor and thiazide diuretic in hypertension management Data retrieved via National Health Insurance Research Database of Taiwan Follow-up period: at least 12 months	17,568 patients newly diagnosed with hypertension aged ≥18 years who were prescribed with FDC (13,176 patients) or free combination (4,392 patients) of renin–angiotensin system inhibitors and thiazide diuretic.	Adherence was measured via PDC.	FDC was associated with better adherence (PDC 58.01% vs. 46.96%; *p* < 0.001) than free combination therapy.
Tilea et al. [77], 2018, Romania Retrospective cross-sectional study	To assess the level of adherence to antihypertensive treatment and analyze how FDCT affects it Family medicine practice in Tirgu Mures, Romania Follow-up period: 48 months	525 patients ≥18 years of age, newly diagnosed with HT, who started with therapy that continued for at least 3 consecutive months (90 on FDCT in the beginning, 173 in the end).	Adherence was measured via prescription records review.	Interventions based on FDC during all 4 years of study showed significantly higher adherence compared to interventions with single active ingredients (*p* = 0.001).
Verma et al. [79], 2018, Germany Retrospective cohort study	To compare clinical outcomes and patient adherence with FDC therapy and multipill therapy Data retrieved via Ontario Drug Benefit database Follow-up period: 5 years	13,350 patients ≥66 years of age who were new users of antihypertensive therapy (6675 on multipill therapy and 6675 on FDCT).	Adherence was measured via the time to the first instance of discontinuation and PDC.	The median time to the first discontinuation of therapy as well as the PDC was higher in FDC group (191 days, 70%) than in multipill group (150 days, 42%; *p* < 0.01).
Lauffenburger et al. [74], 2017, USA Retrospective cohort study	To investigate patterns of antihypertensive therapy initiation and compare adherence and persistence between patients initiating FDC and single-pill therapies Data retrieved via a large national health insurer Follow-up period: 12 months	484,493 patients ≥18 years of age, who initiated an oral antihypertensive medication therapy (78,958 on FDC, 383,269 on single-pill therapy, 22,266 on multipill therapy).	Adherence was measured via PDC.	Patients with FDC therapy were 13% more likely to be adherent than patients on single-pill therapy (RR: 1.13; 95% CIs: 1.11–1.14; *p* < 0.05).
Tung et al. [53], 2017, Taiwan Retrospective cohort study	To compare the clinical outcomes of FDCs and free combinations of ARB and CCB in management of HT Data retrieved via National Health Insurance Research Database of Taiwan Follow-up period: 2.1 years (mean)	5680 hypertensive patients ≥18 years of age, who were prescribed an ARB and a dihydropyridine CCB (1136 on FDC therapy and 4544 on free combination therapy).	Adherence was measured via PDC.	Adherence was higher among patients receiving an FDC compared with the free combination group (PDC ≥80%: 64.97% vs. 56.88%; PDC from 50% to 80%: 22.55% vs. 24.16%; PDC <50%: 12.48% vs. 18.95% (*p* < 0.001)).
Levi et al. [42], 2016, Italy Retrospective cohort study	To compare adherence to FDCT and LDCT in primary care Data retrieved via HS IMS Health LPD, an Italian general practice database Follow-up period: 6 months	6612 hypertensive patients ≥18 years of age, who were treated with olmesartan/amlodipine as an extemporaneous combination or FDC (2090 on extemporaneous combination and 4522 on FDCs).	Adherence was measured via PDC.	55.1% of the patients treated with FDC were found to be highly adherent (PDC >80%), whereas, among patients treated with the extemporaneous combination, only 15.9% were highly adherent (*p* < 0.001).
Sonawane et al. [76], 2016, USA Retrospective cohort study	To compare the adherence of alternative treatment modification strategies and characterize the factors associated with adherence after such modifications Data retrieved via BlueCross BlueShield of Texas commercial claims data Follow-up period: 12 months	5998 hypertensive patients aged ≥18 years who received treatment modifications (1395 on free-pill strategies and 1207 on FDC therapy).	Adherence was measured via PDC.	Adherence for FPC and FDC strategies was 0.67 ± 0.25 and 0.69 ± 0.29, respectively, which was not statistically significant (*p* < 0.05).
Hsu et al. [5], 2015, Taiwan Retrospective cohort study	To compare adherence and persistence in hypertensive patients on FDCT and LDCT among newly diagnosed hypertensive patients Patient data obtained from the National Health Insurance Research Database (NHIRD) Follow-up period: 24 months	7348 newly diagnosed HT patients ≥20 years of age (5725 on FDC therapy and 1623 on free combination therapy).	Adherence was measured via MPR.	Adherence was higher for patients on FDCT than patients on free dosing: 66.6% vs. 63.9% after six months; 52.6% vs. 46.7% after one year; 42.1% vs. 32.5% after two years (all *p* < 0.001).
Machnicki et al. [82], 2015, USA Retrospective cohort study	To assess whether amlodipine/valsartan/hydrochlorothiazide SPC is associated with improved adherence, persistence, and reduced healthcare utilization and costs compared to the FCT Data retrieved using the Truven MarketScan Commercial and Medicare Supplemental Database Follow-up period: 12 months	14,594 hypertensive patients ≥18 years of age (10,800 in single-pill group, 3794 in free combination group).	Adherence was measured via PDC and MPR.	Patients on SPC exhibited higher adherence according to MPR (85.7% vs. 77.0%) and mean PDC (73.8% vs. 60.6%), all *p* < 0.0001.
Degli Esposti et al. [70], 2014, Italy Retrospective cohort study	To investigate the reasons for prescribing polypills and the influence of polypills on adherence in hypertensive patients. Three Italian local health units (patient data retrieved via the Medications Prescription Database) Follow-up period: 6 months	21,008 hypertensive patients ≥18 years of age with a 6-month history of receiving free combination treatment (2395 patients) or polypill treatment (18,613 patients).	Adherence was measured via PDC.	An increased percentage of patients who switched to FDCT were adherent: +24% when coming from a two-pill regime and +42% when coming from single-pill regime (*p* < 0.001).
Tung et al. [78], 2014, Taiwan Retrospective cohort study	To compare the clinical outcomes, healthcare costs, persistence, and adherence of HT treatment with an FDC of amlodipine/valsartan and free-drug combinations of ARB and CCB Data retrieved via the National Health Insurance Research Database (NHIRD) of Taiwan Follow-up period: 15 months	16,505 patients ≥18 years of age with the diagnosis of HT (13,204 in FDC group FDC, 3301 in combination therapy group).	Adherence was measured via PDC.	The FDC group had a significantly higher PDC than the combination therapy group (80.35% vs. 72.57%, *p* < 0.001).
Wang et al. [80], 2014, Taiwan Retrospective cohort study	To assess the effect of single-pill formulations on adherence in hypertensive patients Patient data retrieved from the Taiwanese National Health Insurance database Follow-up period: 12 months	896 patients who switched from free pill combination therapy to FDC therapy of the same compound.	Adherence was measured via MPR.	In patients with low or intermediate preindex adherence (*n* = 729), switching to SPCs resulted in improved MPR (36% difference; 95% CIs: 33%–39%; *p* < 0.001). However, patients with high preindex adherence (*n* = 167) switching to SPCs resulted in a lower MPR (−13% difference; 95% CIs: −17% to −9%; *p* < 0.001).
Xie et al. [81], 2014, USA Retrospective cohort study	To assess what the impact of the pill burden is on adherence in hypertensive patients Data retrieved via health care claims from the MarketScan Commercial and Medicare Supplemental database Follow-up period: 12 months	17,465 hypertensive patients ≥18 years of age, who were prescribed three antihypertensive agents in the form of single-, double- or triple-pill regimens (8516 in single-pill group, 7842 in double-pill group, 1107 in triple-pill group).	Adherence was measured via PDC.	Patients in the double-pill cohort and triple-pill cohort were 55% and 74%, respectively, less likely to be adherent than patients receiving only one pill (*p* < 0.001).
Panjabi et al. [83], 2013, USA Retrospective cohort study	To assess the impact of fixed- versus loose-dose triple-combination therapy on adherence, clinical, and economic outcomes in patients with hypertension Data retrieved from a large US health plan associated with OptumInsight Follow-up period: at least 12 months	16,290 patients initiating triple therapy with an ARB, ACEi, or BB plus amlodipine and hydrochlorothiazide (10,696 on two-pill therapy (FDC + a second pill) and 5594 on a three separate pills therapy).	Adherence was assessed via PDC.	Mean PDC was greater in patients receiving two-pill therapy (ARB cohort: three-pill = 0.41, two-pill = 0.53; ACEi cohort: three-pill = 0.43, two-pill = 0.50; BB cohort: three-pill = 0.42, two-pill = 0.55; *p* < 0.001).
Baser et al. [65], 2011, USA Retrospective cohort study	To compare adherence of valsartan/amlodipine SPC to ARB/CCB multiple-pill free combination Data retrieved via US commercial healthcare insurance claims Follow-up period: 12 months	12,628 hypertensive patients ≥18 years of age (3259 in single-pill group, 9369 in free combination group).	Adherence was measured via PDC.	Patients on SPC were 1.38 times more adherent to their therapy than multiple pill users (95% CIs: 1.24–1.53).
Hussein et al. [72], 2010, USA Retrospective cohort study	To compare the adherence between polypill and two-pill regimen of the same drugs (statin + CCB) Patient data obtained from the Health Plan Claims (US) database Follow-up period: 6 months	35,430 patients ≥18 years of age with a pharmacy claim for single-pill amlodipine/atorvastatin or claims for both a CCB and a statin within any 30-day window in a selection year (patients were categorized into 4 cohorts according to use of CCB and/or statin therapies before the index date and within each cohort based on receiving FDC or multipill therapy).	Adherence was measured via PDC.	Adherence rates were overall higher for polypill groups and varied depending on patients’ previous treatment experiences. The differences in adherence range from no significant difference (OR = 1.00) in naïve patients to significantly higher adherence (OR = 2.81, *p* < 0.001) in the experienced cohort.
Yang et al. [55], 2010, USA Retrospective cohort study	To compare compliance, persistence, health care resource utilization and costs among hypertensive patients on FDCT and LDCT Data retrieved via Thomson Reuters MarketScan Commercial and Medicare Supplemental Databases Follow-up period: 6 months	579,851 patients ≥18 years of age initiating on either of the selected FDC therapies (382,476 patients) or the equivalent free-pill therapies (197,375 patients).	Adherence was measured via MPR.	Patients receiving FDCT showed significantly higher MPR than patients on free-pill therapies (72.8% vs. 61.3%; 95% CI: 11.4%, 11.7%; *p* < 0.05).
Zeng et al. [56], 2010, USA Retrospective cohort study	To assess adherence to ARB/CCB FDC therapy compared with free-pill combination Data retrieved via MedImpact Healthcare Systems database Follow-up period: 12 months	4525 hypertensive patients ≥18 years of age initiating on either of selected FDC (2213 patients) or free-pill therapies (2312 patients).	Adherence was measured via PDC.	Patients in the FDC group were significantly more likely to adherent (OR = 1.90, *p* < 0.001) compared to patients on free combination therapy.
Chapman et al. [68], 2009, USA Retrospective cohort study	To compare the rate of adherence between patients on one polypill and patients with the same drugs in separate pills Data retrieved using PharMetrics Patient-Centric Database Follow-up period: 6 months	4556 hypertensive patients ≥18 years of age prescribed amlodipine who switched to amlodipine/atorvastatin FDC (1139 patients) or added a statin to their amlodipine regimen (3417 patients).	Adherence was measured via PDC.	After 180 days, the follow-up showed that patients on the polypill had a greater improvement in adherence in comparison to multiple pill cohort: 50.8% vs. 44.3% (*p* < 0.001).
Hess et al. [41], 2009, USA Retrospective cohort study	To evaluate medication compliance, persistence and hypertension-related expenditures among patients that switched from FDC to free-combination therapy Data obtained from the Thomson Medstat MarketScan database Follow-up period: 12 months	14,449 patients (7224 switching to free combination therapy and 7225 controls continuing their FDC therapy) were enrolled.	Adherence was measured via MPR.	Adherence among the patients continuing on FDC therapy was 22.1% higher (*p* < 0.001) compared to the patients who switched to free combination therapy.
Brixner et al. [33], 2008, USA Retrospective cohort study	To compare the adherence, persistence and medication costs between single- and multipill drugs Data retrieved via IHCIS National Managed Care Benchmark Database Follow-up period: 12 months	8711 hypertensive patients ≥18 years of age, who were prescribed study drugs in combination and had at least 110 days of recorded data during which no other antihypertensive medications were prescribed before the start of therapy (8510 in FDC group, 561 in multipill group).	Adherence was measured via MPR.	Adherence in patients receiving FDCT was higher compared to the multipill treatment: adherence rates were 64.2% for FDCT and 57.6% for LDCT (*p* < 0.001).
Dickson and Plauschinat [39], 2008, USA Retrospective cohort study	To investigate the difference between FDCs and separate drugs in adherence and total costs Patient data retrieved from South Carolina Medicaid database Follow-up period: 12 months	5704 patients aged 65–100 years who received at least two prescriptions for study drugs in one of the selection years (2336 in FDC group and 3368 in free combination group).	Adherence was measured via MPR.	Adherence was significantly higher in patients receiving FDCT than patients receiving free-dose therapy: 63.4% vs. 49.0% (*p* < 0.0001).
Dickson and Plauschinat [38], 2008, USA Retrospective cohort study	To assess adherence with antihypertensive therapy among African American and White Medicaid patients receiving FDC or free combination therapy Patient data retrieved from the South Carolina Medicaid database Follow-up period: 12 months	4076 patients aged 18–100 years who received at least two prescriptions for study drugs in one of the selection years (3363 in the FDC group and 713 in the free combination group).	Adherence was measured via PDC.	Adherence was significantly higher in patients on FDCT compared to LDCT: 58.6% vs. 48.1% (*p* < 0.05).
Patel et al. [46], 2008, USA Retrospective cohort study	To investigate if the adherence in hypertensive patients is better with an FDC than with multiple pills Patient data retrieved from MedImpact Healthcare Systems Follow-up period: 6 months	4703 patients ≥18 years of age who started a CCB or statin treatment simultaneously or within 30 days (5 cohorts, only one (*n* = 795) receiving polypill therapy).	Adherence was measured via PDC.	After 180 days, the adherence rates of the polypill group were 9%–17% higher than those of other groups (*p* < 0.001) After one year, 63.9% of FDCT patients were adherent, while only 33.1%–43.6% were adherent in the group with the separate pills (*p* < 0.001).
Gerbino and Shoheiber [40], 2007, Italy Retrospective cohort study	To check differences in adherence patterns between an antihypertensive polypill and the drugs taken separately Data retrieved via a pharmacy claims database of a managed care organization in the USA Follow-up period: 12 months	6206 hypertensive patients, who received at least two prescriptions for FDC or double-pill therapy (2839 in FDC group, 3367 in double-pill group).	Adherence was measured via MPR.	Adherence rates were significantly higher in the FDCT group in comparison to the double-pill group: 87.9% vs. 69.2% (*p* < 0.0001).
Diabetes (*n* = 10)				
Rombopoulus et al. [85], 2015, Greece Prospective cohort study	To evaluate the differences in the adherence in DMII patients on FDC and free-dose therapy of the selected drugs Multiple centers in Greece Follow-up period: 24 weeks	659 diabetic patients aged >18 years with inadequate glycemic control with metformin monotherapy (366 on FDC and 293 on free-dose therapy).	Adherence was measured via a questionnaire.	In FDC group, 98.9% of patients were compliant, compared to 84.6% in free-dose group (*p* < 0.005). The odds ratio for FDC vs. free-dose group was 18.9 (95% CIs: 6.2–57.7; *p* < 0.001).
Lokhandwala et al. [86], 2015, USA Retrospective cohort study	To compare persistence, adherence and economic outcomes between diabetic patients using FDC and LDC products Data retrieved via MarketScan Commercial and Medicare Supplemental Databases Follow-up period: 12 months	23,361 patients ≥18 years of age with DMII and one additional oral anti-diabetic prescription of the same regimen (FDC/LDC) as the index prescription; 12,590 on FDCT and 10,771 on LDCT.	Adherence was measured via MPR.	FDC patients had significantly higher rate of adherence than patients on LDCT (OR = 1.28; 95% CIs: 1.20–1.36; *p* < 0.001).
Vittorino Gaddi et al. [84], 2013, Italy Retrospective cohort study	To evaluate antidiabetic drug adherence between MT, LDCT, and FDCT Patient data obtained via the ARNO database Follow-up period: 12 months	169,375 diabetes patients with at least one oral antidiabetic prescription claim (91,816 in MT group, 31,674 in FDCT group and 19,573 in LDCT group; 15.5% were excluded due to therapy switch in the follow-up period).	Adherence was measured via MPR.	Adherence rates were higher in the FDCT group (68.5%) than in LDCT group (60.3%) (*p* < 0.05).
Barner [31], 2011, USA Retrospective cohort study	To compare the adherence and costs between MT, LDCT, and FDCT in the treatment of DMII Data retrieved via Texas Medicaid prescription claims database Follow-up period: at least 12 months	270 patients aged 18–65 years prescribed FDCT with pioglitazone and metformin in post index period and the analogous LDCT or MT in pre index period.	Adherence was measured via MPR.	There was a significant increase in adherence of 8.9% (76.0% to 82.8%) when switching from LDCT to FDCT (*p* = 0.0081).
Thayer et al. [51], 2010, USA Retrospective cohort study	To assess changes in adherence and HbA1c in diabetes patients on different drug regimes Data obtained via two large databases (not specified) Follow-up period: at least 6 months	16,490 patients ≥18 years of age with 1 or more prescription fills for rosiglitazone, a sulfonylurea, or rosiglitazone/glimepiride FDCT during the identification period (patients were grouped according to baseline and follow-up period treatment plan; 2518 switched from mono to dual therapy, 543 from MT to FDCT, 13,145 remained on dual, 284 from dual to FDCT).	Adherence was measured via MPR.	Switching from dual therapy to FDC therapy showed a statistically significant increase in adherence rate (*p* < 0.001).
Cheong et al. [36], 2008, USA Retrospective cohort study	To check the influence of multiple drug regimens (FDCT/dual therapy) on patient adherence Patient data retrieved via the Texas Medicaid prescription claims database Follow-up period: 12 months	22,512 patients aged 22–89 years, who were prescribed an oral antidiabetic FDCT or the analogous dual therapy during the identification period (7750 FDCT users and 14,762 dual therapy users).	Adherence was measured via MPR.	Patients on FDCT had a higher MPR than dual therapy users: 78.6% vs. 77.2% (*p* < 0.001). Patients who switched from dual therapy to FDCT saw an increase in MPR of 12.4%, whereas people who continued dual therapy only saw a rise of 5.1% (*p* < 0.001).
Pan et al. [44], 2008, USA Retrospective cohort study	To compare the patient adherence between single-pill (FDCT) and two-pill regimen Patient data retrieved via the Medstat MarketScan database Follow-up period: 6 months	9170 patients ≥18 years of age prescribed metformin or sulfonylurea or both before July 2000 and both metformin and sulfonylurea concurrently (either separately or FDC) after August 2000 (2275 FDC users and 6895 non-FDC users).	Adherence was measured via MPR.	The adherence to the FDCT in comparison to the two-pill regimen was 12.8% higher (*p* = NA).
Vanderpoel et al. [54], 2005, USA Retrospective cohort study	To observe the changes in adherence rates in patients switching from mono- or dual therapy to a FDCT Data retrieved via pharmacy claims database Follow-up period: 6 months	16,928 patients ≥18 years of age with at least one pharmacy claim for rosiglitazone or metformin during the identification period (patients were grouped according to treatment change from preindex to postindex period; 14,291 remained on mono therapy, 1230 on dual therapy, 931 switched from mono to dual, 349 from mono to FDCT, 127 from dual to FDCT).	Adherence was measured via MPR.	A significant improvement has been observed for patients switching from dual therapy to FDCT (3.5% vs. −1.3%, *p* < 0.005).
Blonde et al. [32], 2003, USA Retrospective cohort study	To check the impact of single-pill drugs on HbA1c values and adherence rates in DMII patients Patient data retrieved via Medco Health Solutions and Quest Diagnostics Follow-up period: 6 months	1421 patients aged 18–80 years who initiated single-pill or multipill therapy and had A1C measurements at baseline and within 76–194 days of initiating combination therapy (471 on multipill therapy and 950 on single-pill therapy).	Adherence was measured via MPR.	Patients were more adherent to the polypill in comparison to two-pill regimen: 84% vs. 76% (*p* < 0.0001).
Melikian et al. [43], 2002, USA Retrospective cohort study	To investigate if adherence is different in diabetes patients with different drug regimens (FDCT, MT, combination therapy) Patient data obtained via pharmacy claims from a pharmacy benefit and medical-management company Follow-up period: 6 months	6502 patients ≥18 years of age who had an index pharmacy claim for an oral antidiabetic and were continuously enrolled in the health plan (4545 receiving metformin MT, 1651 glyburide MT, 219 combination therapy (59 of those switched to FDCT), 87 FDCT).	Adherence was measured via MPR.	For newly diagnosed diabetics, there was no significant difference in adherence between the therapies. Patients switching from combination therapy to FDCT (pill burden reduction) showed a better adherence with FDCT: 71% vs. 87% (*p* < 0.001).
HIV (*n* = 14)				
Langebeek et al. [88], 2014, the Netherlands, Belgium Randomized controlled trial	To investigate the effect of simplified regimens (1 pill/multiple pills) on adherence, life quality and treatment satisfaction 11 different sites in Belgium and the Netherlands Follow-up period: 24 months	120 HIV patients (59 on multipill therapy and 61 on single-pill therapy).	Adherence was measured via Simplified Medication Adherence Questionnaire.	Single pill therapy resulted in better adherence than multipill therapy (*p* = NA).
Arrabal-Duran et al. [90], 2017, Spain Observational prospective study	To data on the effectiveness of switching to an FDC regimen in HIV patients with sustained virological suppression Gregorio Marañon University Hospital, Madrid, Spain Follow-up period: 96 weeks	57 HIV patients whose previous therapy was based on twice-daily therapy regimen and switched to the examined FDC therapy.	Adherence was measured via PDC.	The proportion of patients with adherence <90% improved from 15.5% to 10.4% (*p* = 0.915), when they switched to FDC therapy, but this difference is not statistically significant.
Chen et al. [91], 2016, USA Observational prospective study	To study adherence barriers associated with medication regimen complexity and simplification Patients in Atlanta, Georgia Follow-up period: 6 weeks	750 HIV patients aged ≥18 years receiving antiretroviral therapy (166 patients on FDC, 300 taking single-dose multipill regimen, 284 taking multi-dose multipill regimen).	Adherence was measured via pill count.	A higher number of patients in polypill group (76%) achieved ≥85% adherence compared to both the group taking single-dose (68%) and the group taking multi-dose multipill regimen (66%); *p* < 0.043.
Buscher et al. [34], 2012, USA Prospective cohort study	To study the impact of antiretroviral therapy regimen on adherence in new HIV patients (FDC vs. LDC and once-daily vs. twice-daily dosing) Houston, TX Follow-up period: 18 months	99 newly diagnosed HIV patients (34 on FDCT, 36 on once daily multipill regimen, 29 on twice daily regimen).	Adherence was measured via a 30-day VAS scale.	No significant difference in adherence was seen between the FDCT and LDCT once-daily dosed group (*p* = 0.34).
Airoldi et al. [87], 2010, Italy Prospective cohort study	To check if there is a link between a reduction in pill burden and adherence in HIV patients 6 medical centers in Italy Follow-up period: 6 months	212 HIV patients who switched from multipill to single-pill therapy.	Adherence was measured via VAS.	Adherence increased clinically meaningfully for 1.1% (*p* = 0.01).
Bangsberg et al. [30], 2010, USA Prospective cohort study	To check the influence of a decreased pill burden on adherence in HIV therapy Data obtained via the REACH (The Research on Access to Care in the Homeless) cohort Follow-up period: 6 months	118 HIV patients (47 on single-pill therapy, 57 and 14 on different multipill therapies, respectively).	Adherence was measured via unannounced pill-count.	Adherence was significantly greater for polypills than for multiple pill users (*p* = 0.006).
Santoleri et al. [92], 2018, Italy Retrospective cohort study	To compare adherence between patients receiving single or multiple tablet regimen antiretroviral therapy Hospital Pharmacy of “Santo Spirito” Hospital of Pescara, Italy Follow-up period: 5 years	290 patients who had withdrawn from taking antiretroviral drugs for at least 6 months in the 5-year period (66 on single pill and 227 on multipill (2, 3, 4, or 5 pills daily) therapy).	Adherence was measured via RDD/PDD ratio.	Single pill therapy group had excellent adherence value of 0.98, whereas multiple pill therapy groups had lower adherence levels of 0.92–0.96 during years 1–5 of the study.
Yager et al. [94], 2017, USA Retrospective cohort study	To compare antiretroviral and non-antiretroviral adherence between single and multiple tablet regimens Data retrieved via pharmacy refill records (Upstate New York Veterans’ Healthcare Administration) Follow-up period: 1.1 years for multipill and 2.3 for single-pill therapy	1202 HIV patients ≥18 years of age on ≥3 antiretroviral medications for ≥3 months and available pharmacy refill records (165 patients were on single-pill, 1037 on multiple tablet regimens).	Adherence was measured via MPR.	Adherence was significantly higher for single tablet regimens treatment-naïve recipients (80.8%–15.4%) compared to the multipill therapy (65.9%–21.3%), *p* < 0.001.
Sutton et al. [57], 2016, USA Retrospective cohort study	To evaluate the impact of antiretroviral therapy as a single-tablet regimen or multiple-tablet regimen on outcomes in HIV patients Data retrieved via Veterans Health Administration electronic health record system Follow-up period: at least 60 days	15,602 patients to whom HIV medications were dispensed as single-tablet (6191 patients) or multiple-tablet (9411 patients) during the study period.	Adherence was measured via MPR.	The odds of adherence were approximately two times higher in polypill group than in multiple therapy group (OR, 2.16; 95% CIs: 1.92–2.43; *p* < 0.001).
Sutton et al. [93], 2016, USA Retrospective cohort study	To assess the impact of pill burden in HIV patients receiving single-tablet or multi-tablet regimen on clinical outcomes Data retrieved via South Carolina Medicaid medical and pharmacy paid claims data Follow-up period: at least 60 days	2174 patients aged ≥18 years who were receiving a complete antiretroviral single-tablet (580 patients) or multiple-tablet regimen (1594 patients) for at least 60 days	Adherence was measured via PDC.	Adherence was higher in single-pill than in multiple-pill group (80% vs. 67%, *p* < 0.0001).
Raffi et al. [47], 2015, France Retrospective cohort study	To compare adherence and persistence in HIV adult patients receiving combination ART (cART) as a once-daily single-tablet regimen versus other administration schedules Data retrieved via French National Healthcare Insurance Database Follow-up period: mean 32.8 months	362 patients ≥18 years of age receiving cART reimbursed in selection years (76 on single-tablet regimen, 242 taking >1 pill once daily, 248 having >1 daily intake).	Adherence was measured via pill count.	Better adherence was observed with the polypill in comparison with regimens with >1 daily intake but no difference was observed in comparison with regimens involving >1 pill once daily (mean adherence 89.6% for the polypill, 86.4% for cART with >1 pill once daily and 77.0% for cART with >1 daily intake (*p* < 0.0001)).
Tennant et al. [50], 2014, USA Retrospective cohort study	To compare adherence and virologic outcomes in adult HIV patients on single-tablet or multiple-tablet antiretroviral therapy Patients enrolled in AIDS Drug Assistance Program at two independent clinics in South Carolina and Alabama Follow-up period: mean 22 months (multipill group) and 14 months (single-pill group)	389 HIV patients aged ≥18 years with a documented visit to one of the two clinics and prescribed one of the two examined antiretroviral therapy regimens (165 in single-tablet and 224 on multipill therapy).	Adherence was assessed via MPR and self-reporting.	Median adherence rates were similar in both groups, regardless of the way it was assessed (based on clinic records: 91% and 93% (*p* < 0.14) in single-pill and multipill group, respectively; self-reporting: 100% and 99% (*p* < 0.05) in single-pill and multipill group, respectively). However, the proportion of adherent patients was higher in single-pill therapy group; 61.6% vs. 51.5% (*p* = 0.047; based on clinic records) and 92.8% vs. 85.4% (*p* = 0.0179; based on self-reporting).
Cohen et al. [37], 2013, USA Retrospective cohort study	To compare adherence, healthcare utilization and costs in antiretroviral therapy with once-daily single-tablet regimen to the therapy with two or more pills per day Data retrieved from the MarketScan Medicaid Multi-State Database Follow-up period: at least 60 days	7381 patients (5584 taking two or more pills per day and 1797 on a single-pill therapy) with an HIV diagnosis receiving complete antiretroviral therapy.	Adherence was measured via MPR.	Patients on single-tablet regimens were significantly more likely to reach 95% adherence (*p* < 0.01).
Legoretta et al. [89], 2005, USA Retrospective cohort study	To investigate the influence of pill-burden on adherence in HIV-positive patients Data obtained via 2 databases, West Coast and Southeast state Medicaid Follow-up period: at least 2 months	1427 HIV patients ≥18 years of age, who were newly started on antiretroviral therapy and had at least one prescription refill in the first 60 postindex days (1363 on polypill therapy, 64 on multipill therapy).	Adherence was measured via MPR.	Mean adherence was 85% for polypills, while it was significantly lower (75%) for multiple pills therapy (*p* < 0.001).
LUTS/BHP (*n* = 1)				
Drake et al. [95], 2017, the Netherlands Retrospective cohort study	To compare treatment persistence and adherence with α-blocker plus antimuscarinic combination therapy in men with LUTS/BPH between those prescribed an FDC and those on multipill therapy Data retrieved via the Netherlands IMS LifeLink™ LRx database, which contains data from pharmacies and dispensing (general practices) in the Netherlands Follow-up period: 12 months	1891 patients ≥45 years of age, who received combination therapy with study drugs as FDC or multipill therapy (665 on FDC therapy and 1,226 on multipill therapy).	Adherence was measured via MPR.	Adherence was similar in both groups of patients; 80.0% of the patients on FDC therapy were adherent, while the adherence among patients on α-blocker and antimuscarinic concomitant therapy was 85.8% and 75.2%, respectively (*p* = NA).

FDCT, fixed-dose combination therapy; LDCT, loose-dose combination therapy; FDC, fixed-dose combination; LDC, loose-dose combination; SPC, single-pill combination; MT, monotherapy; MPR, medication possession ratio; RDD/PDD, received daily dose/prescribed daily dose; PDC, proportion of days covered; VAS, visual analog scale; ICD, international classification of diseases; CVD, cardiovascular disease; DMII, diabetes mellitus type II; HIV, human immunodeficiency virus; HT, hypertension; LUTS/BPH, lower urinary tract symptoms associated with benign prostatic hyperplasia; CCB, calcium channel blocker; ACE-I, angiotensin-converting enzyme inhibitor type I; ARB, angiotensin receptor II blocker; ART, antiretroviral therapy; CI, confidence interval; OR, odds ratio; NA, not available.

## Appendix C

**Table A3:** General article information regarding disease, drugs, formulation, and outcomes.

Condition, Reference	Active Ingredients in the Polypill and Free-Pill Combination	Dose (mg)	Outcome on Adherence (+: Improved with Using FDC; −: Decreased with Using FDC; 0: No Difference)
CVD [35]	FPC and FDC: acetylsalicylic acid/simvastatin/ramipril	100 mg/40 mg/2.5 mg or 100 mg/40 mg/5 mg or 100 mg/40 mg/10 mg	+
CVD [59]	FPC and FDC: aspirin/simvastatin/lisinopril/HCTZ	75 mg/40 mg/10 mg/12.5 mg	+
CVD [45]	FPC: various; FDC: aspirin/simvastatin/lisinopril/either atenolol or HCTZ	75 mg/40 mg/10 mg/ 50 mg (atenolol) or 12.5 mg (HCTZ)	+
CVD [48]	FPC: various; FDC: aspirin/simvastatin/lisinopril with either atenolol or HCTZ	75 mg/40 mg/10 mg/ 50 mg (atenolol) or 12.5 mg (HCTZ)	+
CVD [52]	FPC: various; FDC: aspirin/simvastatin/lisinopril and either atenolol or HCTZ	75 mg/40 mg/10 mg/ 50 mg (atenolol) or 12.5 mg (HCTZ)	+
CVD [61]	FPC and FDC: amlodipine/atorvastatin	FPCs: amlodipine 5 and 10 mg; atorvastatin 10, 20, 40, and 80 mg; FDCs 5 mg/10 mg or 5 mg/20 mg or 5 mg/40 mg or 5 mg/80 mg or 10 mg/10 mg or 10 mg/20 mg or 10 mg/40 mg or 10 mg/80 mg	+ or −
Hyperlipidemia [58]	FPC and FDC: ezetimibe/statin	10 mg/ varying dose (statin)	0
Dyslipidemia [63]	FPC: simvastatin + ezetimibe or simvastatin + niacin or lovastatin + niacin); FDC: simvastatin/ ezetimibe or simvastatin/ niacin or lovastatin/ niacin	Not mentioned	+
CVD [62]	FPC: niacin extended-release + lovastatin or simvastatin; FDC: niacin extended-release/ lovastatin	Not mentioned	+
Dyslipidemia [60]	FPC and FDC: niacin/statin	Not mentioned	+
CVD [49]	FPC: CCB + ACEi; FDC: amlodipine besylate/benazepril hydrochloride	Not mentioned	+
HT [75]	FPC: ARB + thiazide; FDC: losartan/HCTZ	FPC: not mentioned; FDC: 50 mg/12.5 mg	0
HT [66]	FPC: various; FDC: olmesartan/amlodipine/HCTZ	20 mg/5 mg/12,5 mg or 40 mg/5 mg/12,5 mg or 40 mg/5 mg/25 mg or 40 mg/10 mg/12,5 mg or 40 mg/10 mg/25 mg	+
HT [73]	FPC and FDC: candesartan or valsartan or telmisartan/amlodipine	ARB (8 mg candesartan or 80 mg valsartan or 40 mg telmisartan)/5 mg amlodipine	+
HT [64]	FPC and FDC: ARB/thiazide diuretic or ARB/CCB	Not mentioned	+
HT [67]	FPC and FDC: ramipril/amlodipine or candesartan/amlodipine	Not mentioned	+
HT [69]	FPC and FDC: perindopril/amlodipine	Not mentioned	+
HT [71]	FPC and FDC: RAS inhibitor/thiazide diuretic	Not mentioned	+
HT [77]	FPC and FDC: various antihypertensive medicines	Not mentioned	+
HT [79]	FPC and FDC: ACEi or ARB/thiazide diuretic	Not mentioned	+
HT [74]	FPC and FDC: various antihypertensive medications	Not mentioned	+
HT [53]	FPC and FDC: ARB/dihydropyridine CCB	Not mentioned	+
HT [42]	FPC and FDC: olmesartan/amlodipine	20 mg/5 mg or 40 mg/5 mg or 40 mg/10 mg	+
HT [76]	FPC and FDC: various antihypertensive drugs	Not mentioned	0
HT [5]	FPC and FDC: ARB/thiazide diuretic	Not mentioned	+
HT [82]	FPC: amlodipine + valsartan + hydrocholorothiazide; FDC: amlodipine/valsartan/HCTZ	Not mentioned	+
HT [70]	FPC and FDC: olmesartan/ amlodipine	Not mentioned	+
HT [78]	FPC: ARB + CCB; FDC: amlodipine/valsartan	Not mentioned	+
HT [80]	FPC and FDC: thiazide diuretic/either ACEi or ARB	Not mentioned	+ or −
HT [81]	FPC and FDC: olmesartan or valsartan/HCTZ/amlodipine	Not mentioned	+
HT [83]	FPC: ARB or ACEi or BB + amlodipine + hydrocholorthiazide; FDC: BB/HCTZ + amlodipine or amlodipine/ARB + HCTZ or ARB/HCTZ + amlodipine or amlodipine/ACEi + HCTZ or ACEi/HCTZ + amlodipine	Not mentioned	+
HT [65]	FPC: ARB + CCB; FDC: valsartan/amlodipine	Not mentioned	+
HT [72]	FPC: CCB + statin; FDC: amlodipine/atorvastatin	Not mentioned	+
HT [55]	FPC: ARB + CCB or ARB + HCTZ, or ACEi + HCTZ; FDC: ARB/CCB or ARB/HCTZ, or ACEi/HCTZ	Not mentioned	+
HT [56]	FPC: ARB + dihydropyridine CCB; FDC: valsartan/amlodipine or amlodipine/olmesartan medoxomil	Not mentioned	+
HT [68]	FPC: amlodipine + another statin; FDC: amlodipine/ atorvastatin	Not mentioned	+
HT [41]	FPC and FDC: ARB/HCTZ or ACE-I/HCTZ or ACEi/CCB	Not mentioned	+
HT [33]	FPC and FDC: valsartan/HCTZ	Not mentioned	+
HT [39]	FPC: CCB + ACEi; FDC: amlodipine/benazepril	Not mentioned	+
HT [38]	FPC: CCB + ACEi; FDC: amlodipine besylate/benazepril hydrochloride	Not mentioned	+
HT [46]	FPC and FDC: amlodipine/atorvastatin or amlodipine/statin or atorvastatin/CCB or CCB/ statin	Not mentioned	+
HT [40]	FPC: CCB + ACEi; FDC: amlodipine/benazepril	Not mentioned	+
DMII [85]	FPC and FDC: vildagliptin/metformin	FPC: 50 mg vildagliptin + 850 mg metformin; FDC: not mentioned	+
DMII [86]	FPC and FDC: various oral antidiabetic drugs	Not mentioned	+
DMII [84]	FPC and FDC: various oral antidiabetics	Not mentioned	+
DMII [31]	FPC and FDC: pioglitazone/metformin	Not mentioned	+
DMII [51]	FPC: thiazolidinedione + sulfonurea; FDC: rosiglitazone/glimepiride	Not mentioned	+
DMII [36]	FPC and FDC: any 2 oral antidiabetic drugs (metformin/glyburide/rosiglitazone…)	Any market-available dose could be included.	+
DMII [44]	FPC and FDC: metformin/sulfonylurea	Not mentioned	+
DMII [54]	FPC and FDC: metformin/thiazolidinedione	2 mg/1000 mg or 4 mg/1000 mg or 1 mg/500 mg or 2 mg/500 mg or 4 mg/500 mg	+
DMII [32]	FPC and FDC: glyburide/metformin	glyburide from 6 to 10 mg/day/ metformin from 893 mg to 1297 mg/day	+
DMII [43]	FPC and FDC: metformin/glyburide	Not mentioned	+ or 0
HIV [88]	FPC: lopinavir/ritonavir + zidovudine/lamivudine; FDC: zidovudine/lamivudine/abacavir	Induction phase: 150 mg lamivudine/300 mg zidovudine twice daily, 400 mg lopinavir/100 mg ritonavir twice daily. Test phase: group 2 kept the same regimen, group 1 switched to 300 mg zidovudine/600 mg lamivudine/600 mg abacavir.	+
HIV [90]	FPC: various; FDC: rilpivirine/emtricitabine/tenofovir disoproxil fumarate	Not mentioned	0
HIV [91]	FPC and FDC: various antiretroviral drugs	Not mentioned	+
HIV [34]	FPC: various; FDC: efavirenz/emtricitabine/tenofovir	Not mentioned	0
HIV [87]	FPC: tenofovir + efavirenz + either emtricitabine or lamivudine; FDC: emtricitabine/tenofovir/efavirenz	Not mentioned	+
HIV [30]	FPC: ritonavir-boosted protease inhibitor + two NRTIs or NNRTI + two NRTIs; FDC: efavirenz/emtricibine/tenofovir	Not mentioned	+
HIV [92]	FPC and FDC: various antiretroviral drugs	Not mentioned	+
HIV [94]	FPC and FDC: various antiretroviral drugs	Not mentioned	+
HIV [57]	FPC and FDC: NRTI/NNRTI/PI/CCR5-antagonist/integrase inhibitor	Not mentioned	+
HIV [93]	FPC and FDC: various antiretroviral drugs	Not mentioned	+
HIV [47]	FPC and FDC: various antiretroviral drugs	Not mentioned	+ or 0
HIV [50]	FPC: protease inhibitor + atazanavir or ritonavir + emtricitabine/tenofivor; FDC: efavirenz/emtricitabine/tenofovir	Not mentioned	0
HIV [37]	FPC: various antiretroviral drugs; FDC: tenofovir/emtricitabine/efavirenz	Not mentioned	+
HIV [89]	FPC and FDC: lamivudine/zidovudine	150 mg/300 mg	+
LUTS/BPH [95]	FPC and FDC: α-blocker/antimuscarinic	Not mentioned	0

FPC, free-pill combination; FDC, fixed-dose combination; HT, hypertension; CVD, cardiovascular disease; DMII, diabetes mellitus type II; HIV, human immunodeficiency virus; LUTS/BPH, lower urinary tract symptoms associated with benign prostatic hyperplasia; CCB, calcium channel blocker; ACEi, angiotensin-converting enzyme inhibitor; ARB, angiotensin receptor II blocker; RAS, renin–angiotensin system; HCTZ, hydrochlorothiazide; BB, beta-blocker; NRTI, nucleoside/nucleotide reverse transcriptase inhibitor; NNRTI, nonnucleoside/nucleotide reverse transcriptase inhibitor; PI, protease inhibitor; CCR5, chemokine receptor 5.
